# Measurement properties of cervical joint position error in people with and without chronic neck pain

**DOI:** 10.1371/journal.pone.0292798

**Published:** 2023-10-12

**Authors:** Ahmad AlDahas, Valter Devecchi, Janet A. Deane, Deborah Falla

**Affiliations:** 1 Centre of Precision Rehabilitation for Spinal Pain (CPR Spine), School of Sport, Exercise and Rehabilitation Sciences, College of Life and Environmental Sciences, University of Birmingham, Birmingham, United Kingdom; 2 Department of Physical Therapy, Al-Sabah Medical Hospital, Ministry of Public Health, Kuwait; King Khalid University, SAUDI ARABIA

## Abstract

**Background:**

People with chronic neck pain (CNP) often present with impaired neck proprioception. The most widely used clinical test for assessing neck proprioception is cervical joint position sense which measures joint position error (JPE). This clinical test is typically performed using a laser pointer to examine the accuracy of returning to a neutral head position (NHP) or target head position (THP) following active neck movements. The aim of this study was to determine the measurement properties of JPE using a laser pointer when tested in people with and without CNP under a variety of different testing conditions (i.e., different movement directions, sitting versus standing, NHP versus THP).

**Methods:**

Forty-three participants (23 asymptomatic and 20 with CNP) underwent neck proprioception testing, returning to a NHP and THP in both sitting and standing positions (six trials for each test). A laser pointer was secured on the participant’s forehead and inertial measurement unit (IMU) sensors were placed beneath the laser pointer and at the level of the spinous process of the seventh cervical vertebra. Both the absolute and the constant JPE were assessed.

**Findings:**

For the asymptomatic participants, good reliability (ICC: 0.79) was found only for right rotation of the THP task in sitting. In standing, good reliability (ICC: 0.77) was only found in flexion for the THP task. In standing, good reliability (ICC: 0.77) was only found for right rotation of the THP for the absolute JPE and left rotation (ICC: 0.85) for the constant error of the NHP task. In those with CNP, when tested in sitting, good reliability was found for flexion (ICC: 0.8) for the absolute JPE and good reliability (ICC range: 0.8–0.84) was found for flexion, extension, and right rotation for the constant JPE. In standing, good reliability (ICC range: 0.81–0.88) was found for flexion, and rotation for the absolute JPE. The constant JPE showed good reliability (ICC: 0.85) for right rotation and excellent reliability (ICC: 0.93) for flexion. Validity was weak to strong (r range: 0.26–0.83) and moderate to very strong (r range: 0.47–0.93) for absolute and constant error respectively, when tested in sitting. In standing, the validity was weak to very strong (0.38–0.96) for the absolute JPE and moderate to very strong (r range: 0.54–0.92) for the constant JPE.

**Conclusion:**

The reliability of the measure of JPE when tested in sitting and standing in both groups showed good reliability, but not for all movements. The results of the current study also showed that the laser pointer correlated well with the Noraxon IMUs, but not for all movements. The results of the current study support the use of the JPE using a laser pointer in clinical and research settings.

## Introduction

Chronic neck pain (CNP), is a leading cause of disability worldwide [1]; it is ranked as the 4^th^ leading cause of disability and 21^st^ in overall burden of disease [2]. A common observation in people with CNP is a disturbance in neck proprioception which may be triggered by pain, effusion, trauma, or fatigue, factors which can affect the discharge of the muscle spindles resulting in disrupted proprioception [3]. Neck proprioception is most commonly assessed using the joint position sense test which measures joint position error (JPE). JPE is defined as the error in repositioning the head to a neutral head position (NHP) or to a target head position (THP) without the use of visual cues [4]. In the NHP test, the participant starts from a NHP, performs an active neck movement then attempts to return to the same neutral position [5], while in the THP test, the assessor or the participant selects a target position [5].

In a clinical environment, neck proprioception is commonly assessed with a laser pointer mounted on the patient’s head combined with a wall target [3]. Although the JPE test is commonly used in clinical practice and in research, there is still insufficient knowledge of the measurement properties of this test, including the reliability, validity, and responsiveness of the measure. Some studies have assessed the measurement properties of the JPE assessed using a laser pointer (e.g. [6–11]). However, a variety of testing protocols were used for the assessment of the reliability of the JPE test in terms of movements tested, type of error assessed (absolute versus), type of JPE task, and testing position. For example, Goncalves and Silva [11], investigated the NHP and THP tests but in right and left rotation only. Pinsault et al. [6] evaluated the NHP task in asymptomatic participants and evaluated both absolute and variable errors whereas Roren et al. [7], tested the reliability of the NHP task after returning from right and left rotation analysing absolute error only. Jorgensen et al. [8] positioned the laser at the back of the participants’ heads, while the previously mentioned studies mounted the laser on their forehead. All the previously mentioned studies were limited to testing of right and left rotation. Meanwhile, Dugailly et al. [9] tested flexion, extension, and right and left rotation in five asymptomatic participants at low and high movement speeds and included only the constant error in their analysis. Also, the number of testing trials was not standardised between studies. All previous studies tested JPE in a sitting position, and no study was identified testing the reliability of the JPE test in standing position using a laser pointer, however, two studies were identified testing the measurement properties of the JPE test in a standing using other tools [12, 13]. Overall, it is evident that a more comprehensive evaluation is required to establish the reliability of the JPE when tested in different movement directions, different testing positions and when considering both absolute and constant error. There is also limited research testing the criterion-related validity of the JPE test using a laser pointer in sitting. Studies that have tested this measurement property were limited to tests of right and left rotation [14, 15], while Dugailly et al. [9] tested rotation as well as flexion and extension. In addition, no study has investigated the criterion-related validity of the JPE using a laser pointer measured in standing.

Based on the apparent need to evaluate the measurement properties of the JPE in both people with and without CNP in a range of different movement directions, testing positions (sitting and standing), and conditions (NHP and THP), the aims of this study were as follows: 1. to determine the reliability of the JPE test when assessed to a NHP and THP following active neck flexion, extension, and right and left rotation in people with and without CNP when performed in sitting and standing positions, 2. to determine the criterion-related validity of the JPE test when assessed to a NHP and THP following active neck flexion, extension, and right and left rotation in asymptomatic people when performed in sitting and standing positions, 3. to compare the JPE between groups, and 4. to compare JPE when tested in sitting and standing positions.

## Methods

### Study design

A between-day test-retest (intra-rater) reliability and criterion-related validity study was carried out on people with and without CNP. The COnsensus-based Standards for the selection of health status Measurement INstruments (COSMIN) guidelines of study designing checklist [16] was used to report this study. Testing was carried out by a single rater who has experience in COSMIN guidelines for reporting studies of measurement properties and received extensive training on measuring JPE using a laser pointer and inertial measurement units (IMUs). The study was carried out in a motion analysis laboratory at the Centre of Precision Rehabilitation for Spinal Pain (CPR) Spine, University of Birmingham, United Kingdom. The study received full ethical approval from the ethics committee at the University of Birmingham (ERN_21–0618). All participants provided written informed consent. Data collection was carried out from December 2021 to May 2022. Participants identities and personal information were stored in a locked room at the University, and no one has access to their information.

### Participants

All participants were recruited from the staff and student population at the University of Birmingham, United Kingdom. Participants were initially screened via email through a health screening questionnaire to ensure their eligibility for the study.

#### Inclusion and exclusion criteria

An inclusion criterion for both groups was adults aged between 18–55 years with a restriction on the upper limit to limit potential degenerative changes of the spine with older age [17]. Asymptomatic participants had to be free of neck pain. People with CNP had to have experienced neck pain for at least 3 months [14] with at least mild pain (≥4 out of 10 on a Numerical Rating Scale) [18] or at least 10 out of 100 on the Neck Disability Index [14]. The Numerical Rating Scale is a reliable and valid self-administered questionnaire to measure the level of pain intensity [19]. The Neck Disability Index is a reliable and valid method to measure disability in people with neck pain [20]. Exclusion criteria for both groups were neck surgery, vestibular disorders, and Covid-19 related symptoms in the last 14 days according to the requirements of the University of Birmingham.

### Testing procedure

Testing of the asymptomatic participants was spread over four testing days to minimise fatigue during testing and to reduce learning effects. Testing in sitting always took place before testing in standing. The NHP test was carried out first followed by the THP test, but the order of tested movements was randomised using the following website (https://www.random.org/lists/). The first session consisted of reliability session 1 and validity testing in sitting. The second session consisted of reliability session 1 and validity testing in standing. After 14 days, as recommended by COSMIN, the participants were asked to come for two additional testing days for reliability testing in sitting (session 3) then standing (session 4). In each testing session, the order of tested movements was randomised to minimise learning effects.

People with CNP were asked to attend two testing sessions only. They carried out the NHP test only to reduce fatigue and potential changes in their pain intensity due to repeated testing. CNP participants were tested in the same way as asymptomatic participants, testing in sitting always took place before standing but the order of tested movements was randomised. Testing sessions were arranged as follows: the first session consisted of reliability testing in sitting and standing and the second session consisted of reliability testing in sitting and standing. The second session took place 14 days after day 1, as recommended by COSMIN.

All participants were instructed not to perform any strenuous exercise or activity during the period between testing sessions that could potentially aggravate their pain or lead to fatigue.

At the beginning of the first session, participants with CNP were asked to complete the Dizziness Handicap Inventory questionnaire, and Tampa Scale of Kinesiophobia questionnaire. Asymptomatic participants completed the Tampa Scale of Kinesiophobia questionnaire only. People with CNP also completed the Tampa Scale of Kinesiophobia and the Dizziness Handicap Inventory questionnaires in session 2 to determine whether there was any change between sessions. The Dizziness Handicap Inventory is a questionnaire that is sub-grouped into three domains (functional, emotional, and physical) that assesses dizziness over 25 items [21]; Dizziness Handicap Inventory scores range from 0 to 100 [22]. Dizziness Handicap Inventory scores can be further sub-divided according to the sub-groups of the questionnaire into 28 points for physical, 36 points for functional, and 36 points for emotional [22]. The Tampa Scale of Kinesiophobia is a questionnaire that contains 17 items that assess the person’s fear of movement and movement-related behaviour [23]; Tampa Scale of Kinesiophobia scores range from 17–68, where higher scores mean higher degrees of Kinesiophobia [23].

A laser pointer specifically designed for clinical testing of proprioception (Tracker ^TM^, USA) was fixed on the participant’s forehead and additionally, IMUs (Research PRO IMU, Noraxon, USA) with a sampling rate of 100 Hz were used simultaneously with the laser pointer to investigate the criterion-related validity of the two testing tools. The IMUs by Noraxon are reliable and valid for measuring body kinematics [24]. One IMU sensor was fitted on the forehead beneath the laser pointer via double-sided tape and one sensor was fitted over the neck at the level of the seventh cervical spinous process. Prior to data collection, the IMUs were calibrated, and the starting position was set at 0°.

**Fig 1:**
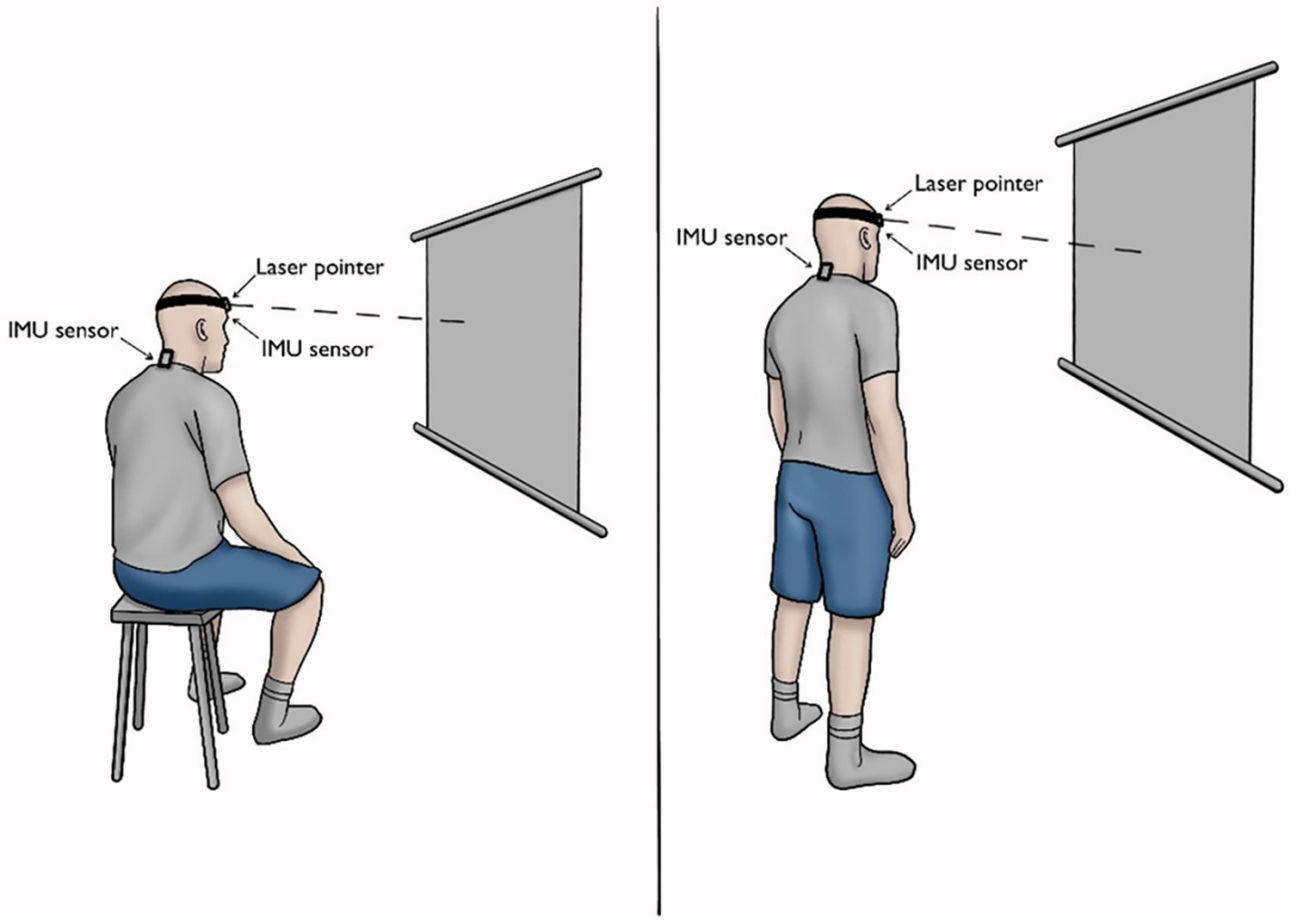
An illustration of the testing procedure performed in sitting and standing.

The participant sat/stood in a comfortable position in front of an A2-sized paper that was positioned 90 cm in front of them at eye level (Fig 1). Tests of JPE to a NHP and a THP were carried out. During the NHP tests, participants were asked to stay in a neutral position for a few seconds then the rater marked this position on the paper as the starting position (NHP). The participant moved their head away from the start position in their full range of motion at a comfortable pace and then attempted to reposition the head back to the starting point as accurately as possible, whereas for the THP, the participant was asked to reposition the head to a target position (approximately mid-range) predetermined visually by the investigator (AA).

For all JPE tests performed, flexion, extension, right rotation, and left rotation were tested in a random order in both sitting and standing. Participants performed one familiarisation trial with their eyes open and after that, they were asked to close their eyes, perform the active movement and repeat the movement six times; six trials have been recommended to assess spinal proprioception [25, 26]. They were instructed to keep their eyes closed during all six repetitions. A one-minute rest was given after each trial. The movement was evaluated in the primary plane only.

### Data analysis

The difference between the starting position (zero) and the point of return in the plane of movement was measured in centimetres [3, 7, 14] and then converted to degrees using this formula angle = tan^-1^ [error distance/90 cm] [7]. The average of the six trials was calculated and taken forward for data analysis. Both absolute and constant errors were determined. The absolute error was defined as the mean of total deviation from the target ignoring the positive and negative values [27]. The constant error was defined as the mean of total deviation from the target considering positive and negative values [27].

To analyse the IMU data, the two waveforms of cervical flexion/extension and right rotation/left rotation were exported from myoRESEARCH software (Noraxon, USA) to MATLAB where they were analysed with customised script. The waveform referring to the primary plane of movement was considered. The starting position was visually identified from the angle and velocity waveforms, and it was recognised by the steady position just before starting the movement. The return position was considered as the one at the end of the recording. The JPE was then computed as the difference between the start and return position. Both absolute and constant JPE were computed as previously described.

### Statistical analysis

G*Power (version 3.1.9.4) was used to determine the required sample size for the evaluation of reliability and validity. The sample size for the reliability analysis was determined based on a 0.05 significance level, true reliability exceeding 0.7, and power of 0.8, resulting in a required sample of ≥19 participants [28]. For validity, a sample of ≥13 participants is required to detect a correlation of 0.7, with a level of significance 0.05, and power of 0.8 [29]. Therefore, a target of at least 20 participants was set for each group accounting for possible dropouts.

Data analysis was carried out using SPSS (version 27, IBM). The Shapiro-Wilk test was used to assess the normality of data distribution. Since the data were not normally distributed, the Mann Whitney U test was used to analyse the difference in JPE between people with and without CNP and between sitting and standing positions. Also, the Mann Whitney U test was used to analyse any differences in the demographic data between the asymptomatic and the CNP group. Significance was set at (p≤0.05). For the reliability analysis, the ICC, standard error of measurement (SEM), and Bland Altman limits of agreement were assessed. The two-way mixed model ICC (3, K) with absolute agreement and average measure was used for analysis. The following criteria was used for ICC interpretation: <0.5 = poor, 0.5–0.74 = moderate, 0.75–0.9 = good, and >0.91 = excellent [30]. The SEM was obtained from the repeated measure analysis of variance (ANOVA) as the square root of the mean square residual using the following formula SEM = √σ 2 residual [31]. For Bland Altman plots interpretation of good agreement, the majority of points must rely within 95% limits of agreement with even distribution of points on both sides and a mean difference closer to zero [32].

For criterion validity, the two-tailed Pearson’s product-moment correlation coefficient (r) was used. The following criteria was used for validity interpretation; 0.1–0.39 = weak, 0.4–0.69 = moderate, 0.7–0.89 = strong, 0.9–1 = very strong [33].

## Results

Twenty-three asymptomatic participants (8 men, 15 women) and 20 people with CNP (8 men, 12 women) participated. The demographic data of the participants are summarised in Table 1. Statistical analysis showed no significant differences between the groups in terms of their age, height, and weight however, the Tampa Scale of Kinesiophobia scores were significantly greater in those with CNP compared to asymptomatic controls.

**Table 1:** Participant demographic data presented as means and standard deviations.

	Asymptomatic	CNP	P-value for group difference
**Gender**	8 males, 15 females	8 males, 12 females	-
**Age, mean (SD)**	25.66 (6.85)	25.9 (4.75)	P = 0.64
**Height (cm), mean (SD)**	170.13 (7.47)	162.48 (26.01)	P = 0.15
**Weight (kg), mean (SD)**	68.56 (15.98)	78.61 (29.07)	P = 0.45
**TSK, mean (SD)**	32.69 (7.8)	40.1 (5.9)	P = 0.001*
**NDI, mean (SD)**	-	27.6 (6.34)	-
**NRS, mean (SD)**	-	4.05 (1.87)	-
**DHI, mean (SD)**	-	25.7 (15.01)	-

TSK = Tampa Scale of Kinesiophobia. NDI = Neck Disability Index. NRS = Numerical Rating Scale. DHI = Dizziness Handicap Inventory. CNP = chronic neck pain. The Mann-Whitney U test was used to assess significance.

* = P-value ≤0.05.

There was one dropout from the asymptomatic group due to Covid-19 related symptoms, thus data for tests performed in standing are presented for 22 asymptomatic and 20 people with CNP. Fifteen asymptomatic participants and 19 people with CNP completed the reliability study whereas for validity analysis, data for 15 asymptomatic participants were included in sitting data analysis and 14 in standing data analysis due to technical difficulties with one recording. There was no difference in the Tampa Scale of Kinesiophobia (p = 0.44) and the Dizziness Handicap Inventory (p = 0.95) questionnaires between sessions 1 and 2 in those with CNP.

### Differences in JPE between people with and without chronic neck pain

In sitting, there was a significant difference between groups in absolute JPE for flexion, extension, and left rotation (Table 2), while JPE for right rotation was not significant between groups. No differences were observed between groups for the constant error apart from the constant JPE in left rotation (Table 2).

**Table 2:** Differences in absolute and constant JPE between asymptomatic participants and people with CNP when tested in sitting.

Absolute error, mean (SD)				Constant error, mean (SD)			
Movement	Asymptomatic	CNP	P-value	Movement	Asymptomatic	CNP	P-value
**Flexion**	2.09 (0.63)	4.3 (3.33)	0.001*	**Flexion**	-0.02 (1.78)	0.73 (5.18)	0.84
**Extension**	2.23 (0.82)	4.8 (2.69)	0.001*	**Extension**	1.13 (2.23)	1.28 (4.9)	0.78
**Right rotation**	2.46 (2.02)	3.53 (2.36)	0.072	**Right rotation**	0.84 (2.53)	0.47 (4.06)	0.3
**Left rotation**	1.86 (0.96)	3.68 (2.11)	0.001*	**Left rotation**	-0.14 (1.81)	-2.62 (3.07)	0.002*

* = P-value ≤0.05. CNP = chronic neck pain

When tested in standing, there was a significant difference in the absolute JPE for flexion, extension, and right rotation (Table 3), while JPE following left rotation was not significant between groups. No differences were observed between groups for the constant JPE (Table 3).

**Table 3:** Differences in absolute and constant JPE between asymptomatic participants and people with CNP when tested in standing.

Absolute error, mean (SD)				Constant error, mean (SD)			
Movement	Asymptomatic	CNP	P-value	Movement	Asymptomatic	CNP	P-value
**Flexion**	2.07 (1.14)	4.93 (3.12)	0.001*	**Flexion**	-0.16 (1.9)	1.15 (5.44)	0.42
**Extension**	2.19 (0.92)	4.06 (2.68)	0.001*	**Extension**	0.94 (1.66)	-0.04 (4.63)	0.096
**Right rotation**	1.96 (1.18)	3.41 (2.18)	0.013*	**Right rotation**	0.92 (1.64)	0.19 (3.92)	0.19
**Left rotation**	2.34 (1.6	3.06 (1.91)	0.13	**Left rotation**	-0.27 (2.28)	0.37 (3.06)	0.48

* = P-value ≤0.05. CNP = chronic neck pain.

### Differences in JPE between sitting and standing

For the asymptomatic participants, there was no significant difference in JPE between sitting and standing for both the absolute and constant errors (Table 4). In people with CNP, there was only a significant difference in JPE between sitting and standing positions for the constant error of left rotation (Table 5) thus the measures were largely the same regardless of the testing position.

**Table 4:** Differences in JPE when tested in sitting and standing positions for the asymptomatic participants.

Absolute error, mean (SD)				Constant error, mean (SD)			
Movement	Sitting	Standing	P-value	Movement	Sitting	Standing	P-value
**Flexion**	2.09 (0.63)	2.07 (1.14)	0.33	**Flexion**	-0.02 (1.78)	-0.16 (1.9)	0.82
**Extension**	2.23 (0.82)	2.19 (0.92)	0.96	**Extension**	1.13 (2.23)	0.94 (1.66)	0.76
**Right rotation**	2.46 (2.02)	1.96 (1.18)	0.48	**Right rotation**	0.84 (2.53)	0.92 (1.64)	0.48
**Left rotation**	1.86 (0.96)	2.34 (1.6)	0.45	**Left rotation**	-0.14 (1.81)	-0.27 (2.28)	0.63

**Table 5:** Differences in JPE when tested in sitting and standing positions for the participants with CNP.

Absolute error, mean (SD)				Constant error, mean (SD)			
Movement	Sitting	Standing	P-value	Movement	Sitting	Standing	P-value
**Flexion**	4.37 (3.33)	4.93 (3.12)	0.41	**Flexion**	0.73 (5.18)	1.15 (5.44)	0.75
**Extension**	4.8 (2.69)	4.06 (2.68)	0.37	**Extension**	1.28 (4.9)	-0.04 (4.63)	0.26
**Right rotation**	3.53 (2.36)	3.41 (2.18)	0.97	**Right rotation**	0.47 (4.06)	0.19 (3.92)	0.84
**Left rotation**	3.68 (2.11)	3.06 (1.91)	0.27	**Left rotation**	-2.62 (3.07)	0.37 (3.06)	0.003*

* = significance (P-value ≤0.05).

### Reliability

For the asymptomatic group, the absolute error for the NHP task when performed in sitting showed poor to moderate reliability (ICC range: 0.4–0.66) with a SEM range of 0.46°-0.91° and poor to good reliability (ICC range: -0.27–0.79) for the THP task with a SEM range of 0.53°-1.22° (Table 6). For the constant error, poor to good reliability (ICC range: 0.28–0.76) was observed with a SEM range of 0.8°-1.76° for the NHP task and poor to good reliability (ICC range: 0.08–0.77) with a SEM range of 0.93°-1.77° for the THP task (Table 6).

**Table 6:** Reliability analysis of JPE measured from asymptomatic participants performing the NHP and THP tasks in sitting.

Movement	Absolute error ICC (95% CI)	SEM	Constant error ICC (95% CI)	SEM
**Flexion (NHP)**	0.4 (-0.88–0.8)	0.84	0.28 (-1.31–0.76)	1.76
**Extension (NHP)**	0.66 (0.08–0.8)	0.91	0.52 (-0.18–0.83)	1.57
**Right rotation (NHP)**	0.56 (-0.37–0.85)	0.57	0.71 (0.17–0.9)	0.8
**Left rotation (NHP)**	0.64 (-0.12–0.88)	0.46	0.76 (0.29–0.92)	0.86
**Flexion (THP)**	-0.18 (03.1–0.62)	0.76	0.77 (0.29–0.92)	0.93
**Extension (THP)**	0.04 (-2.27–0.69)	1.22	0.26 (-1.34–0.75)	1.8
**Right rotation (THP)**	0.79 (0.26–0.93)	0.53	0.62 (-0.16–0.87)	1.51
**Lefty rotation (THP)**	-0.27 (-3.36–0.58)	1.24	0.08 (-1.87–0.7)	1.77

ICC = Intraclass correlation coefficient. CI = Confidence interval. SEM = Standard error of measurement. NHP = neutral head position. THP = target head position. SEM = standard error of measurement

When the tests were performed in standing, the absolute JPE showed moderate reliability (ICC range: 0.5–0.74) for the NHP task with a SEM range of 0.48°-0.76° and poor to good reliability (ICC range: 0.21–0.77) for the THP task with a SEM range of 0.51°-1.38° (Table 7). The constant JPE showed poor to good reliability (ICC range: 0.21–0.85) with a SEM range of 0.72°-1.11° for the NHP task and poor to moderate reliability (ICC range: 0.23–0.6) for the THP task with a SEM range of 0.92°-1.55° (Table 7).

**Table 7:** Reliability analysis of JPE measured from asymptomatic participants performing the NHP and THP tasks in standing.

Movement	Absolute error ICC (95% CI)	SEM	Constant error ICC (95% CI)	SEM
**Flexion (NHP)**	0.68 (0.01–0.89)	0.5	0.67 (0.02–0.89)	1.01
**Extension (NHP)**	0.5 (-0.4–0.83)	0.76	0.74 (0.3–0.92)	1.03
**Right rotation (NHP)**	0.5 (-0.52–0.83)	0.6	0.21 (-1.58–0.74)	1.11
**Left rotation (NHP)**	0.74 (0.22–0.91)	0.48	0.85 (0.56–0.95)	0.72
**Flexion (THP)**	0.33 (-1.1–0.78)	1.38	0.55 (-0.37–0.85)	1.55
**Extension (THP)**	0.21 (-1.49–0.74)	1.07	0.6 (-0.1–0.86)	0.92
**Right rotation (THP)**	0.77 (0.3–0.92)	0.51	0.23 (-1.23–0.74)	1.42
**Lefty rotation (THP)**	0.42 (-0.86–0.81)	0.58	0.6 (-0.22–0.86)	1.03

ICC = Intraclass correlation coefficient. CI = Confidence interval. SEM = Standard error of measurement. NHP = neutral head position. THP = target head position.

For those with CNP when tested in sitting, the absolute JPE showed poor to good reliability (ICC range: 0.43–0.8) and a SEM range of 0.59°-2.34° and poor to good reliability (ICC range: -0.09–0.82) with a SEM range of 2.09°-3.43° for the constant error (Table 8). In standing, the absolute JPE showed poor to good reliability (ICC range: 0.19–0.87) with a SEM range of 0.98°-2.64° and moderate to excellent reliability (ICC range: 0.52–0.93) with a SEM range of 1.82°-4° for the constant error (Table 9). The Bland Altman limits of agreement are presented in S1 File.

**Table 8:** Reliability analysis of JPE measured from people with CNP when performing the NHP task in sitting.

Movement	Absolute error ICC (95% CI)	SEM	Constant error ICC (95% CI)	SEM
**Flexion**	0.8 (0.48–0.92)	2.02	0.84 (0.59–0.93)	2.73
**Extension**	0.43 (-0.51–0.78)	2.34	0.8 (0.5–0.92)	2.66
**Right rotation**	0.57 (-0.11–0.83)	1.65	0.82 (0.53–0.93)	2.09
**Left rotation**	0.51 (-0.24–0.81)	0.59	-0.09 (-1-0.51)	3.43

ICC = Intraclass correlation coefficient. CI = Confidence interval. SEM = Standard error of measurement.

**Table 9:** Reliability analysis of JPE measured from people with CNP performing the NHP task in standing.

Movement	Absolute error ICC (95% CI)	SEM	Constant error ICC (95% CI)	SEM
**Flexion**	0.88 (0.69–0.95)	1.38	0.93 (0.81–0.97)	1.82
**Extension**	0.19 (-1.08–0.69)	2.64	0.52 (-0.19–0.81)	4
**Right rotation**	0.81 (0.53–0.92)	1.22	0.85 (0.61–0.94)	2.17
**Left rotation**	0.87 (0.67–0.95)	0.98	0.6 (-0.03–0.84)	2.43

ICC = Intraclass correlation coefficient. CI = Confidence interval. SEM = Standard error of measurement.

### Criterion validity

When tested in sitting, the absolute JPE showed weak to strong correlations (r range: 0.39–0.83) between measures for the NHP task and weak to moderate correlations (r range: 0.26–0.69) for the THP task. For the constant JPE, the NHP task showed moderate to very strong correlations (r range: 0.55–0.93) and moderate correlations (r range: 0.47–0.68) for the THP task (Table 10).

**Table 10:** Criterion-validity analysis performed for the JPE measured from asymptomatic participants for the NHP and THP tasks.

Movement	Sitting (n = 15)		Standing (n = 14)	
	Absolute error	Constant error	Absolute error	Constant error
**Flexion (NHP)**	0.65	0.73	0.95	0.94
**Extension (NHP)**	0.39	0.55	0.75	0.92
**Right rotation (NHP)**	0.83	0.93	0.73	0.78
**Left rotation (NHP)**	0.57	0.65	0.89	0.92
**Flexion (THP)**	0.26	0.68	0.45	0.54
**Extension (THP)**	0.4	0.57	0.96	0.64
**Right rotation (THP)**	0.34	0.55	0.6	0.83
**Left rotation (THP)**	0.69	0.47	0.38	0.54

NHP = Neutral head position. THP = Target head position.

For the tests performed in standing, the absolute JPE showed strong to very strong correlations (r range: 0.73–0.95) for the NHP task and weak to very strong correlations (r range: 0.38–0.96) for the THP task. For the constant JPE, strong to very strong correlations (r range: 0.78–0.94) between measures were observed for the NHP task and a moderate to strong correlations (r range: 0.54–0.83) were observed for the THP task (Table 10).

## Discussion

This study investigated the measurement properties (reliability and criterion validity) of the cervical JPE test when performed in sitting and standing in people with and without CNP. Both absolute and constant errors were included in the analysis. The results show that the JPE is reliable, but not for all movements. In addition, the JPE measurements assessed with a laser pointer correlated well with the measurements taken by the IMUs but again, not for all movements. JPE was significantly higher in people with CNP when compared to asymptomatic participants when tested in both sitting and standing, but mainly for the measurement of the absolute error. In most tasks there was no significant difference between the JPE measured in sitting and standing, apart from the constant JPE measured following left rotation.

### Differences in JPE between people with and without chronic neck pain

In the current study when tested in sitting, the absolute JPE for the asymptomatic participants 2.09°-2.46° and 3.53°-4.8° for the CNP group; significant between group differences were observed for flexion, extension, and left rotation. In standing, the absolute JPE for the asymptomatic participants was 1.96°-2.34° and 3.06°-4.93° for CNP group; significant between group differences were observed for flexion, extension, and right rotation. Errors ranging from ≥3°-4° have been suggested as a threshold for indicating proprioceptive deficits on the cervical position sense test [34]. Pooled results from a previous systematic review by de Zoete et al. [35] found that people with idiopathic neck pain repositioned their head with an error 2.2°-9.8°, while asymptomatic participants repositioned the head with an error 1.66°-5.1°. Thus the results of the current study are in line with previous studies that have reported some differences in JPE when comparing asymptomatic people to those with non-traumatic neck pain [36–39].

Some studies have noted differences depending on the movement direction tested. For instance, Kristjansson et al. [38] reported significant group differences in JPE following active rotation, Revel et al. [36] reported significant between group differences for flexion, extension, right and left rotation, Cheng et al. [39] reported significant between group differences for flexion and extension, and Rix and Bagust [37] reported significant between group differences for flexion only. In the current study, significant between group differences were observed for the absolute error when tested in sitting for flexion, extension, and left rotation with right rotation almost reaching statistical significance. Whilst all of these studies evaluated JPE in sitting, it should be noted that several differences exist between studies including the number of trials, the equipment used to assess JPE, the type of neck pain complaint (e.g., WAD, non-specific neck pain) and the level of patient reported pain and disability.

No previous studies have compared JPE between people with and without CNP when tested in standing. In the current study, we observed a significant between group difference in the absolute JPE for flexion, extension, and right rotation when tested in standing, while JPE following left rotation was not significant between groups. No differences were observed between groups for the constant JPE. thus overall, between group differences were largely the same regardless of whether testing performed in sitting or standing.

When comparing withing group differences between sitting and standing positions, we showed no significant difference in JPE between the testing positions for either group apart from left rotation in those with CNP participants which showed a significant difference for the constant error in standing. Kim and Shin [40] investigated differences of JPE in sitting versus standing when returning from flexion, extension, and lateral flexion but only in asymptomatic people. They reported a significant difference in JPE between sitting and standing but only after returning from extension only. The authors argued that during sitting, the neck extensors are more active than in standing and concluded that this may have led to a significant difference between testing positions. Overall, however, it seems that major differences in JPE do not exist when testing is conducted in sitting versus standing.

### Reliability of JPE when assessed in sitting

The Bland Altman LoA plots showed that most points lied between the 95% CI indicating that there was no systematic bias. This was anticipated as the rater took extensive training on the tools used.

In the asymptomatic participants, when performing the NHP task, the absolute error after returning from flexion showed poor reliability (ICC: 0.4) while extension, right and left rotation showed moderate reliability (ICC range: 0.56–0.66). Goncalves and Silva [11] performed the NHP task but only returning from right and left rotation showing good reliability (ICC range: 0.75–0.8), which is superior to our results. Pinsault et al. [6] also tested the reliability of the NHP and showed moderate to good reliability (ICC range: 0.52–0.81) for the absolute error. Our results are similar to those reported by Roren et al. [7] where they showed moderate reliability (ICC: 0.68) for right and left rotation. However, they assessed within-day intra-rater reliability while we examined between-day intra-rater reliability. For THP, only one previous study was identified which is the one by Goncalves and Silva [11] which tested the reliability of the THP after returning from right and left rotation only showing moderate to good reliability (ICC range: 0.55–0.76) while in the current study, the reliability was poor to good (ICC range: -0.27–0.79).

In the current study we also examined the reliability of the constant error for the NHP and THP tasks. Only one earlier study which tested the reliability of constant JPE after returning from flexion, extension, and right and left rotation was identified [9]. In this study, asymptomatic participants performed the tasks at fast and slow speeds and at 90cm and 180cm distances from the target. When comparing their results obtained with the target at 90cm distance and for movement performed at a slow speed (i.e., comparable to the task performed in our study), JPE following rotation, flexion, and extension showed poor reliability (ICC range: 0.22–0.47). Our results were higher than this at least for extension (ICC: 0.52), and right and left rotation (ICC range: 0.71–0.76). Several differences between the studies exist including sample size (n = 5 versus n = 15 in the current study) and the position of the laser pointer. When considering the results for people with CNP, good reliability (ICC: 0.8) was observed for flexion, whereas extension showed poor reliability (ICC: 0.43) and right and left rotation showed moderate reliability (ICC range: 0.51–0.57). Goncalves and Silva [11] also examined people with CNP but examined right and left rotation only showing moderate to good (ICC range: 0.61–0.8) reliability.

Interestingly, the reliability of JPE was better when testing our participants with CNP than for the asymptomatic group. This could potentially be explained by fatigue due to more extensive testing of the asymptomatic participants especially since previous studies have confirmed the detrimental effect of fatigue on neck proprioception in the cervical spine [41, 42]. Boredom during data collection may have also impacted on the results [9]. Overall, the wide range of reliability results, which spanned from poor to good, needs to be considered when interpreting changes in JPE e.g., after an intervention.

### Reliability of JPE when assessed in standing

To the best of our knowledge, this is the first study to test the measurement properties of the JPE test using a laser pointer when tested in standing. In asymptomatic participants, the reliability of the measure was moderate (ICC range: 0.5–0.74) and poor to good (ICC range: 0.21–0.77) for the absolute and constant errors respectively for the NHP test. For the THP, the reliability was poor to good (ICC range: 0.21–0.77) and poor to moderate (ICC range: 0.23–0.6) for the absolute and constant error respectively. For those with CNP participants, the reliability was poor to good (ICC range: 0.19–0.88) and moderate to excellent (ICC range:0.52–0.93) for the absolute and constant error respectively for the NHP test. Similar results have been reported by Strimpakos et al. [12] albeit using a motion analysis device and not a laser pointer. Time-interval and the number of testing trials are methodological flaws that existed in the study by Strimpakos et al. [12]. Time-interval in their reliability investigation was one week, which is not recommended by COSMIN guidelines [43], which may also indicate that there was a learning effect by the participants. Number of testing trials in their investigation was three per flexion, bilateral rotation, and bilateral bending, however, at least 6 trials are recommended when testing proprioception [44]. When comparing the results of the reliability investigation in sitting vs standing in CNP participants (Tables 8 and 9), the results in standing were superior to sitting. The participants sat in a comfortable position of their choosing instead of strictly upright sitting, which may have caused an increase in the activity of the neck muscles during sitting position [40], thus affecting the results.

### Standard error of measurement

The closer the measurement error is to zero the more reliable the measure is. In the asymptomatic people, in sitting, the measurement error of the absolute NHP showed less error than the absolute THP. Similarly, in standing, the absolute JPE showed less measurement error when compared to absolute THP. In people with CNP, the measurement error was relatively high when compared to asymptomatic participants. This was expected as people with CNP exhibit proprioceptive deficits. In sitting, the absolute JPE showed an error of 0.59°-2.34°. This was low if compared to absolute JPE in standing (0.98°-2.64°).

Alahmari et al. [5] showed a measurement error of 1.78°-1.88° and 1.45°-2.45° for NHP and THP tasks respectively, which is lower than the measurement error in the current study. This can be explained by the movements tested and the population recruited for the study. Extension NHP was assessed in sitting only, whereas THP was assessed after returning from 50% of flexion, extension, bilateral lateral flexion, and bilateral rotation were evaluated in sitting and supine. In the current study we evaluated flexion, extension, and bilateral rotation in sitting and standing and we reported absolute and constant errors also. In their study, the time interval between testing sessions was ≤ 3 working days while in the current study it was 1 week. During this time, pain symptoms could have changed which could affect the results. Lower measurement errors were also reported by Goncalves and Silva [11] for NHP and THP tasks, however, their assessment was limited to the assessment of right and left rotation only.

### Criterion validity

There was a wide range of correlation seen between the measures of JPE with the laser pointer versus the IMUs with correlations ranging from only weak to very strong correlation. The limits of agreement plots revealed that most points were between the 95% CI indicating no systematic bias.

The results obtained when validity was assessed in the sitting position are comparable with previous studies. Roren et al. [7] compared the laser pointer with an ultrasound technique testing only right and left rotation. Their results showed very strong levels of validity (r = 0.95), but only for the absolute error. Chen and Treleavan [14] correlated the laser pointer measure to the 3-Space Fastrak when testing right and left rotation. Their results showed strong levels of validity (r = 0.87) and only for the absolute error. Wibault et al. [15] compared a laser pointer to a CROM device when testing right and left rotation in people with cervical radiculopathy. Their results revealed moderate to very strong validity (ICC: 0.43–0.91). However, importantly these previous studies only investigated the absolute JPE to a NHP following right and left rotation. In contrast, the current study has considered JPE following flexion, extension, right and left rotation for both NHP and THP tests and considered both absolute and constant error, and as a result has revealed more variability in the degree of validity. This variability of correlations is suggested to be from the differences in standard deviation between the two testing devices. The higher the standard deviation, the higher the variability in the results. Another factor that could explain this variability is the human error as the error obtained from the laser is measured by hand, which is subject to error, and it is being compared to a gold standard. Sample size, time taken for data collection, and fatigue are all possible reasons for these differences in correlations.

### Methodological considerations

This research was carried out following COSMIN guidelines [16], which is considered a strength of this study. The average of 6 trials was used to assess cervical proprioception as recommended previously [44]. However, the large number of trials and tests could have led to fatigue and may have affected the results. The testing in this study was carried out by one tester only which is a further limitation of this study.

## Conclusion

This study indicates that the JPE test could be used to differentiate between people with and without CNP. When performed using a laser pointer, the JPE test showed to be reliable and valid but not all movements. Fatigue could be a reason for these poor results. we recommend to re-assess the measurement properties of the NHP alone to minimise potential fatigue.

## Supporting information

S1 File Bland Altman limits of agreement plots. (DOCX)

S2 File Demographic data of asymptomatic participants (total). (XLSX)

S3 File Demographic data of validity testing performed in sitting. (XLSX)

S4 File Demographic data of validity testing performed in standing. (XLSX)

S5 File Demographic data for asymptomatic participants for reliability testing. (XLSX)

S6 File Reliability data of asymptomatic participants performed in sitting. (XLSX)

S7 File Reliability data of asymptomatic participants performed in standing. (XLSX)

S8 File Sitting vs standing data of asymptomatic participants. (XLSX)

S9 File Validity data performed in sitting. (XLSX)

S10 File Validity data performed in standing. (XLSX)

S11 File Demographic data of CNP participants (total) and reliability. (XLSX)

S12 File Asymptomatic vs CNP data performed in sitting. (XLSX)

S13 File Asymptomatic vs CNP data performed in standing. (XLSX)

S14 File Reliability data of CNP participants performed in sitting. (XLSX)

S15 File Reliability data of CNP participants performed in standing. (XLSX)

S16 File Sitting vs standing data of CNP participants. (XLSX)
