# Supplementary material for: Measurement properties of cervical joint position error in people with and without chronic neck pain
Source: PLoS One. 2023 Oct 12;18(10):e0292798. doi: 10.1371/journal.pone.0292798 (PMC10569517; doi:10.1371/journal.pone.0292798)
Supplement: S1 File — (DOCX) [file pone.0292798.s001.docx]

**Reliability**

**Bland Altman plots of JPE in sitting position (asymptomatic participants)**

Bland Altman plots for the limits of agreement for the absolute JPE measured in sitting show that most of the scores lie between the 95% confidence interval with mean differences 0.001-0.63 for NHP (Figure 1) and -0.05-0.57 for THP (Figure 2).


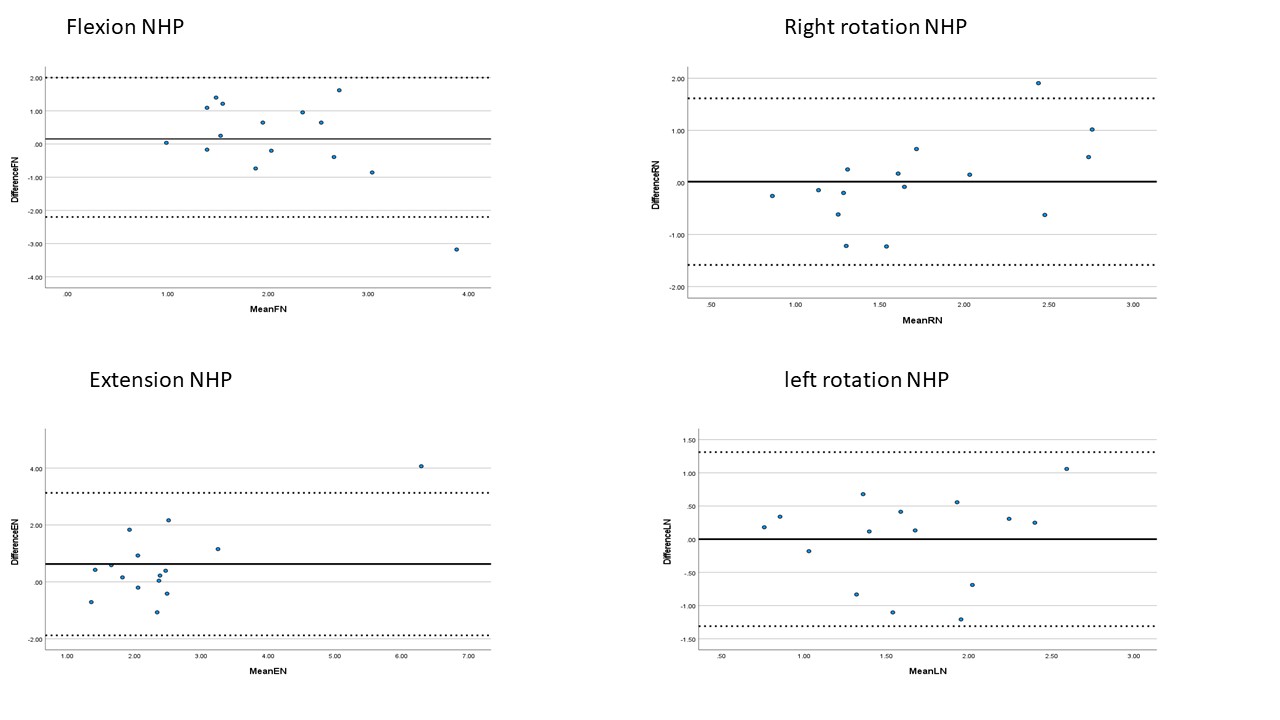


Figure 1, Bland Altman plots for intra-rater reliability of absolute error for flexion direction, extension, and right and left rotation for neutral head position (NHP) task in sitting. Limits of agreement are presented as the dotted lines with the mean difference illustrated by the black line.


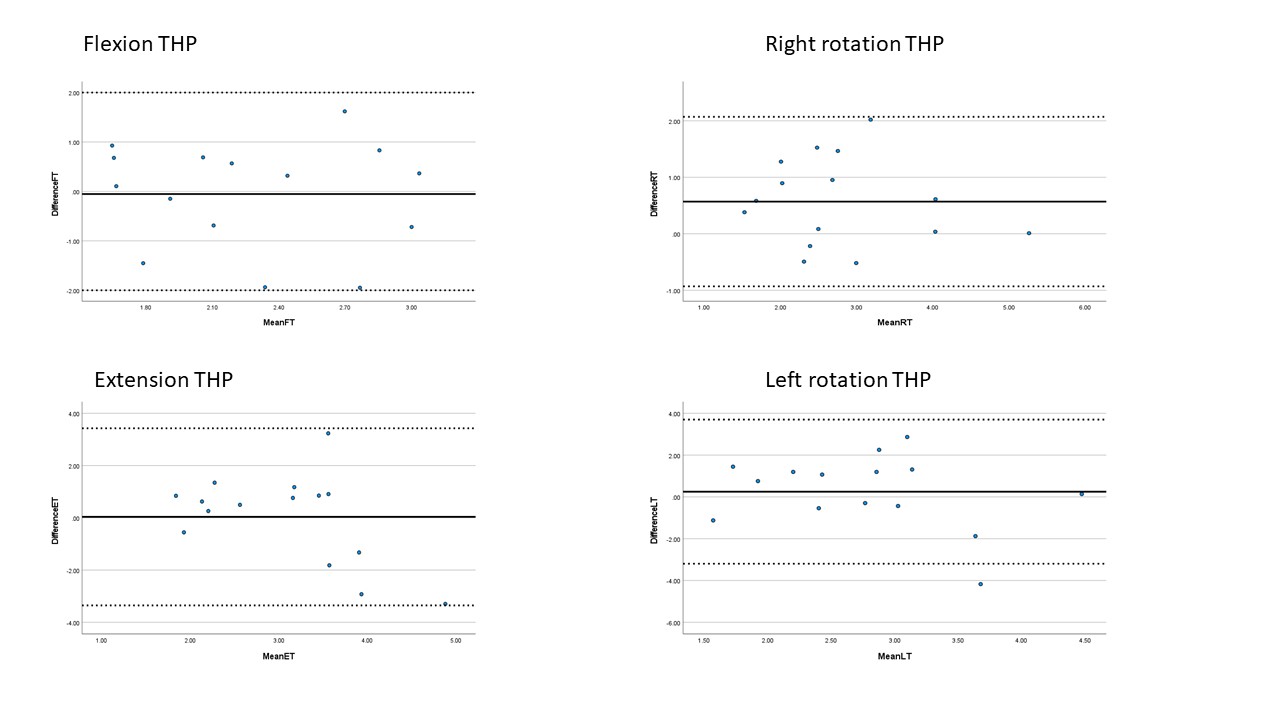


Figure 2, Bland Altman plots for intra-rater reliability of absolute error for flexion direction, extension, and right and left rotation for target head position (THP) task in sitting. Limits of agreement are presented as the dotted lines with the mean difference illustrated by the black line.

Bland Altman plots for the limits of agreement for the constant JPE measured in sitting show that most of the scores lie between the 95% confidence interval with mean differences -0.2-1.41 for NHP (Figure 3) and -0.47-0.38 for THP (Figure 4).


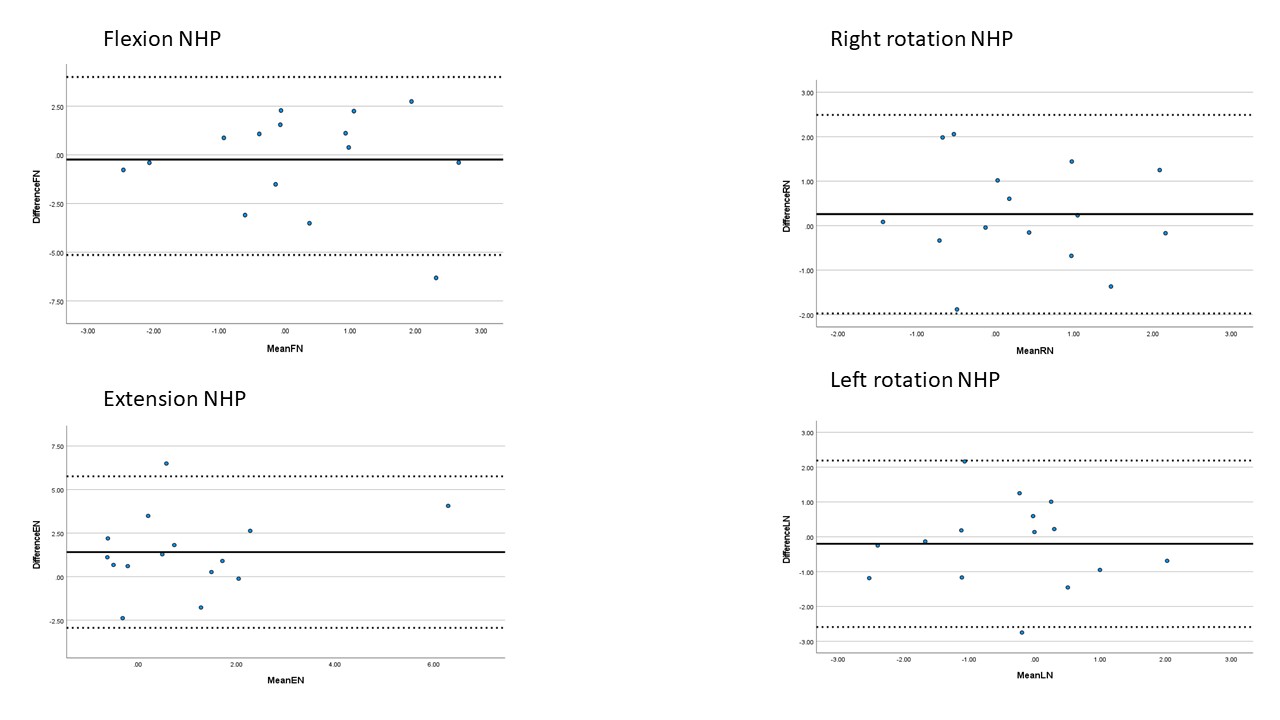


Figure 3, Bland Altman plots for intra-rater reliability of constant error for flexion direction, extension, and right and left rotation for neutral head position (NHP) task in sitting. Limits of agreement are presented as the dotted lines with the mean difference illustrated by the black line.


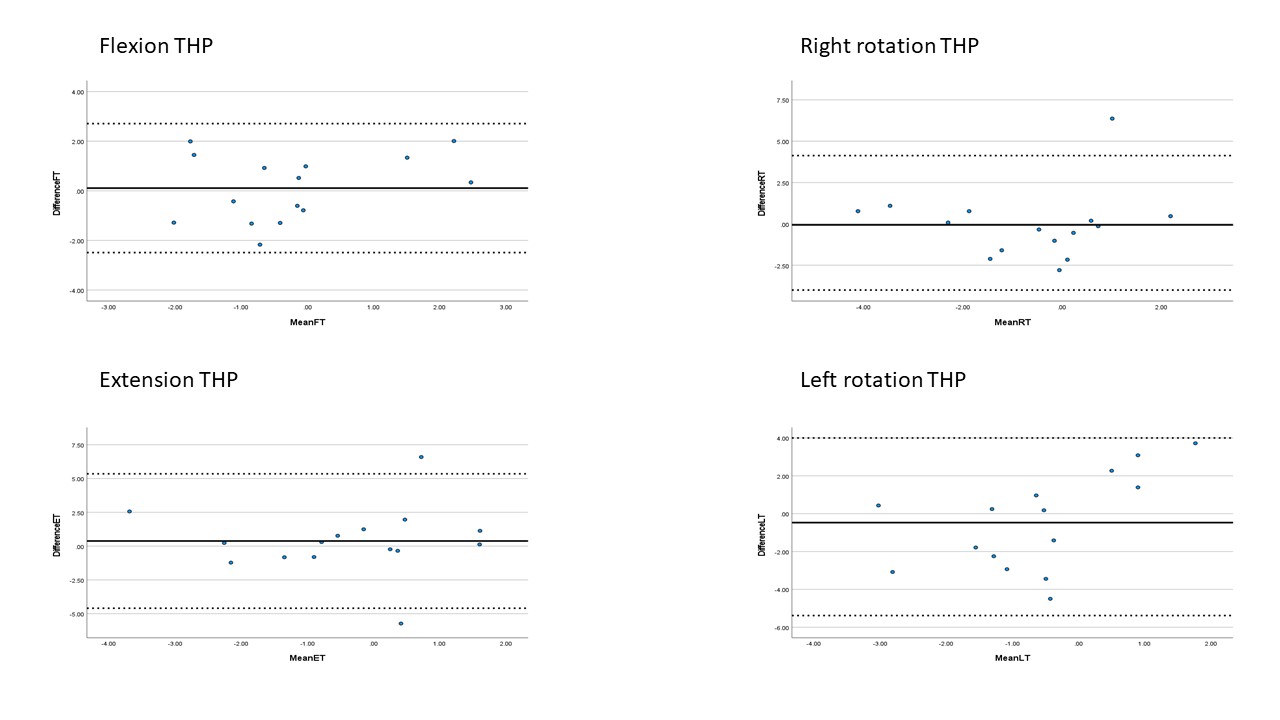


Figure 4, Bland Altman plots for intra-rater reliability of constant error for flexion direction, extension, and right and left rotation for target head position (THP) task in sitting. Limits of agreement are presented as the dotted lines with the mean difference illustrated by the black line.

**Bland Altman plots of JPE in standing position (asymptomatic participants)**

Bland Altman plots for the limits of agreement for absolute JPE measured in standing show that most of the scores lie between the 95% confidence interval with mean differences -0.3-0.11 for NHP (Figure 5) and 0.05-0.28 for THP (Figure 6).


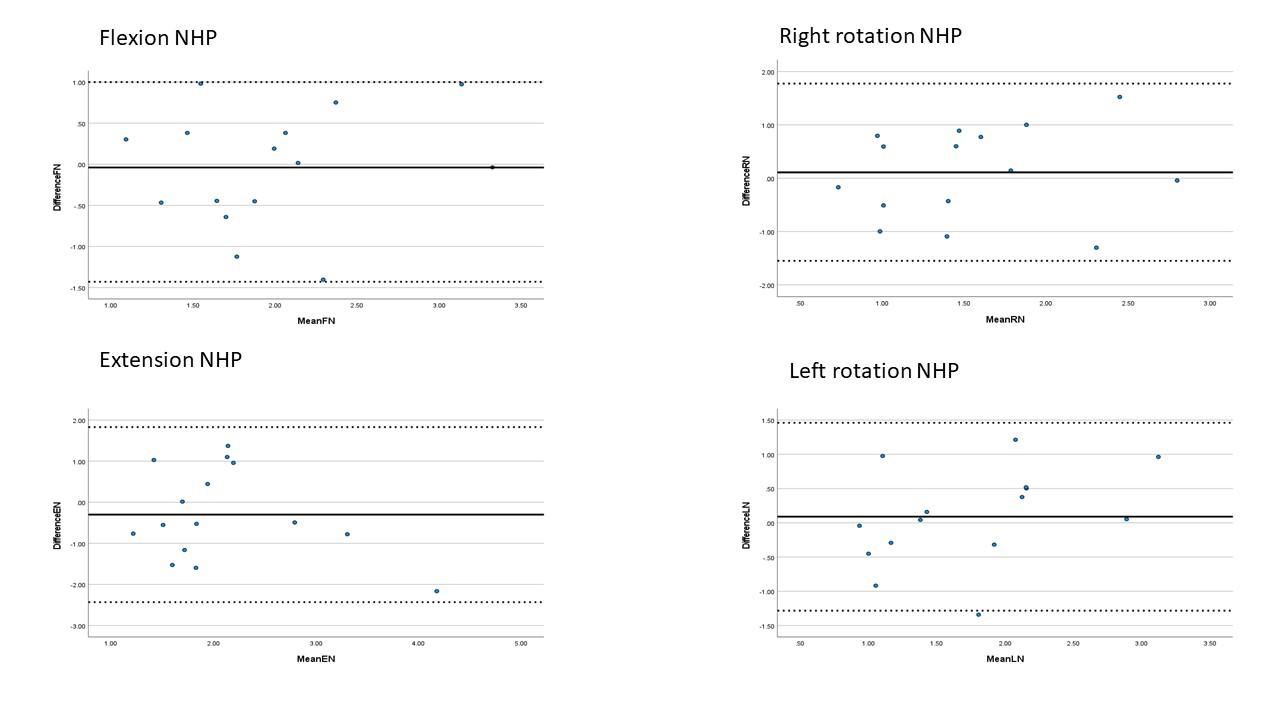


Figure 5, Bland Altman plots for intra-rater reliability of absolute error for flexion direction, extension, and right and left rotation for neutral head position (NHP) task in standing. Limits of agreement are presented as the dotted lines with the mean difference illustrated by the black line.


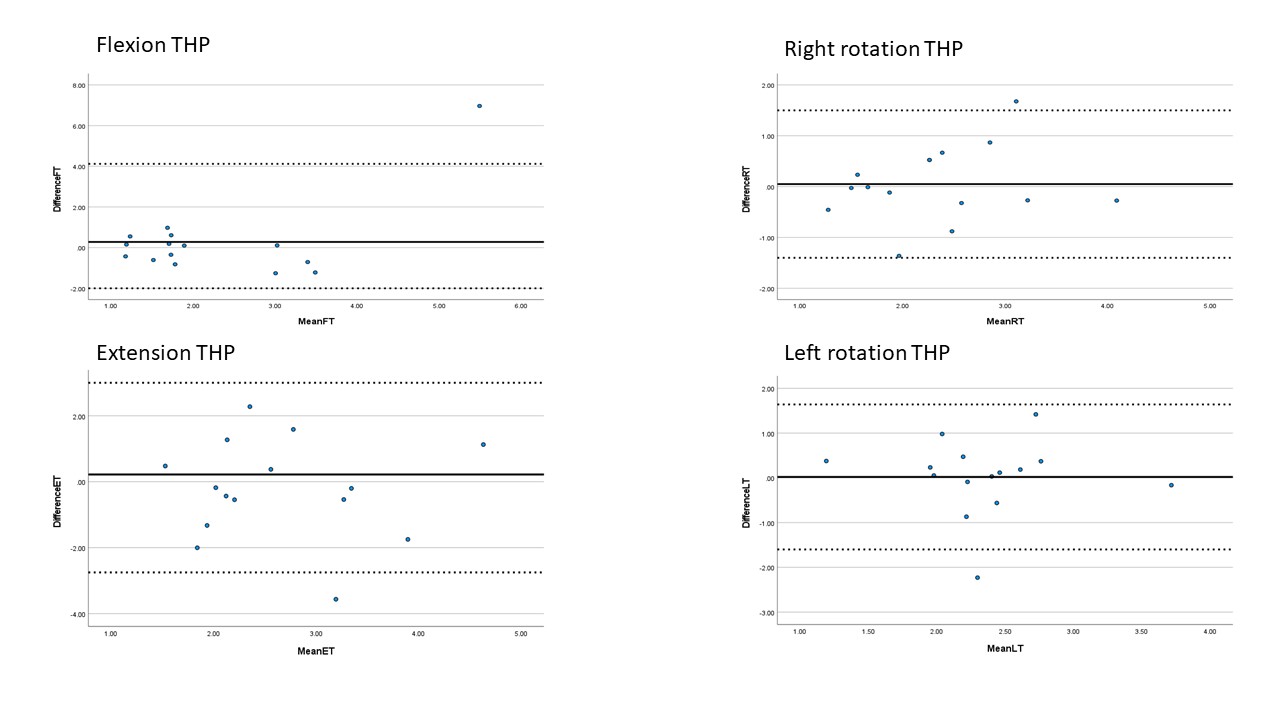


Figure 6, Bland Altman plots for intra-rater reliability of absolute error for flexion direction, extension, and right and left rotation for target head position (THP) task in standing. Limits of agreement are presented as the dotted lines with the mean difference illustrated by the black line.

Bland Altman plots for the limits of agreement for constant JPE measured in standing show that most of the scores lie between the 95% confidence interval with mean differences 0.04-0.23 for NHP (Figure 7) and -0.53-1.06 for THP (Figure 8).


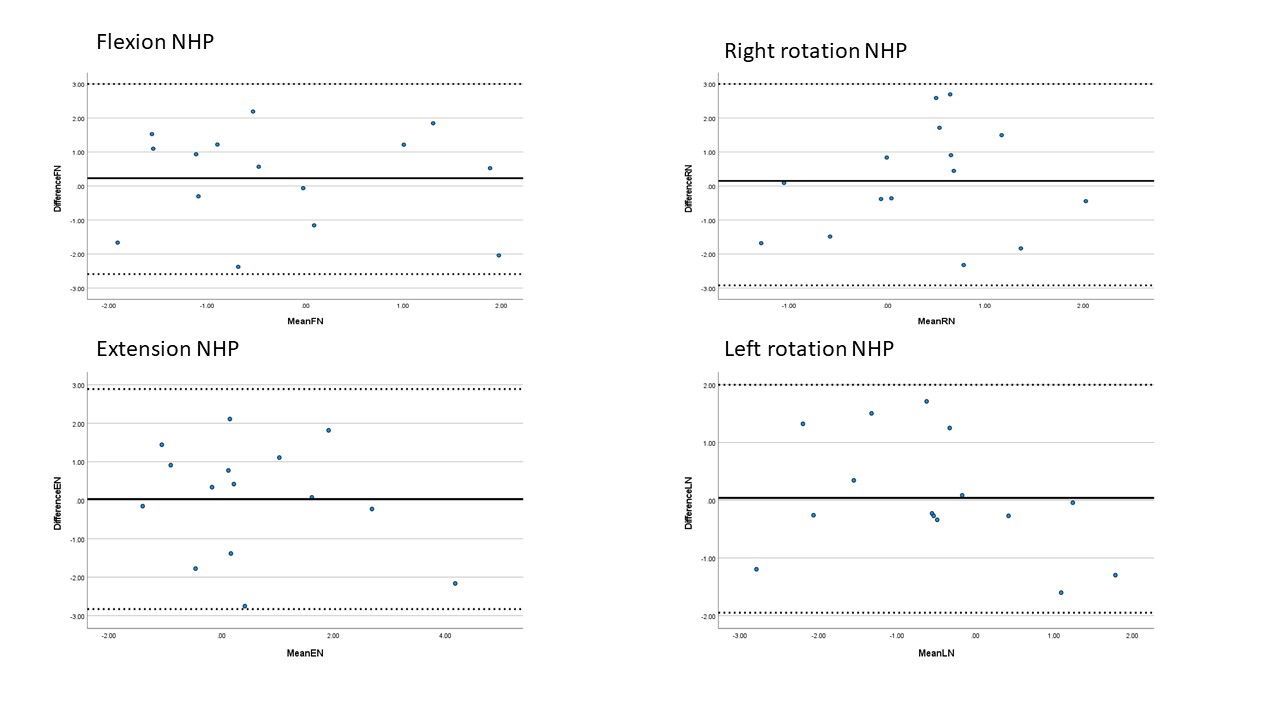


Figure 7, Bland Altman plots for intra-rater reliability of constant error for flexion direction, extension, and right and left rotation for neutral head position (NHP) task in standing. Limits of agreement are presented as the dotted lines with the mean difference illustrated by the black line.


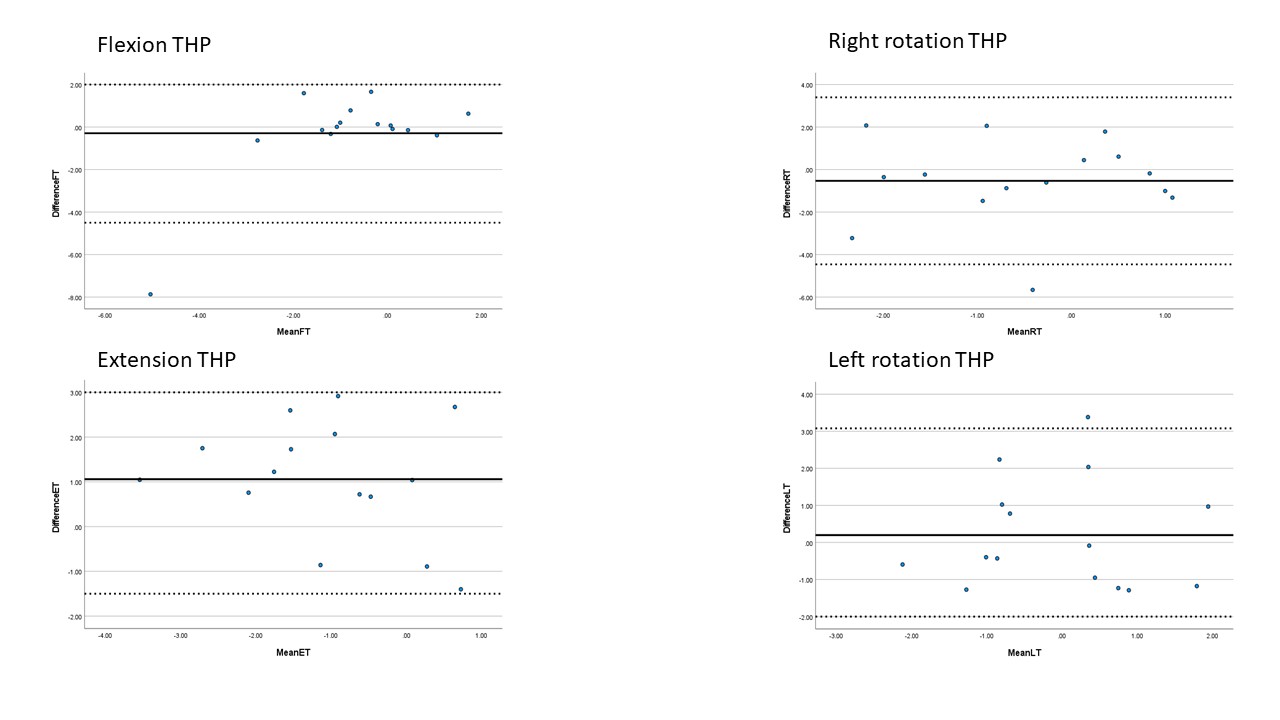


Figure 8, Bland Altman plots for intra-rater reliability of constant error for flexion direction, extension, and right and left rotation for target head position (THP) task in standing. Limits of agreement are presented as the dotted lines with the mean difference illustrated by the black line.

**Bland Altman plots for JPE in sitting (CNP participants)**

Bland Altman plots for the limits of agreement for absolute JPE measured in sitting show that most of the scores lie between the 95% confidence interval with mean differences -0.27-0.48 for NHP (figure 9).


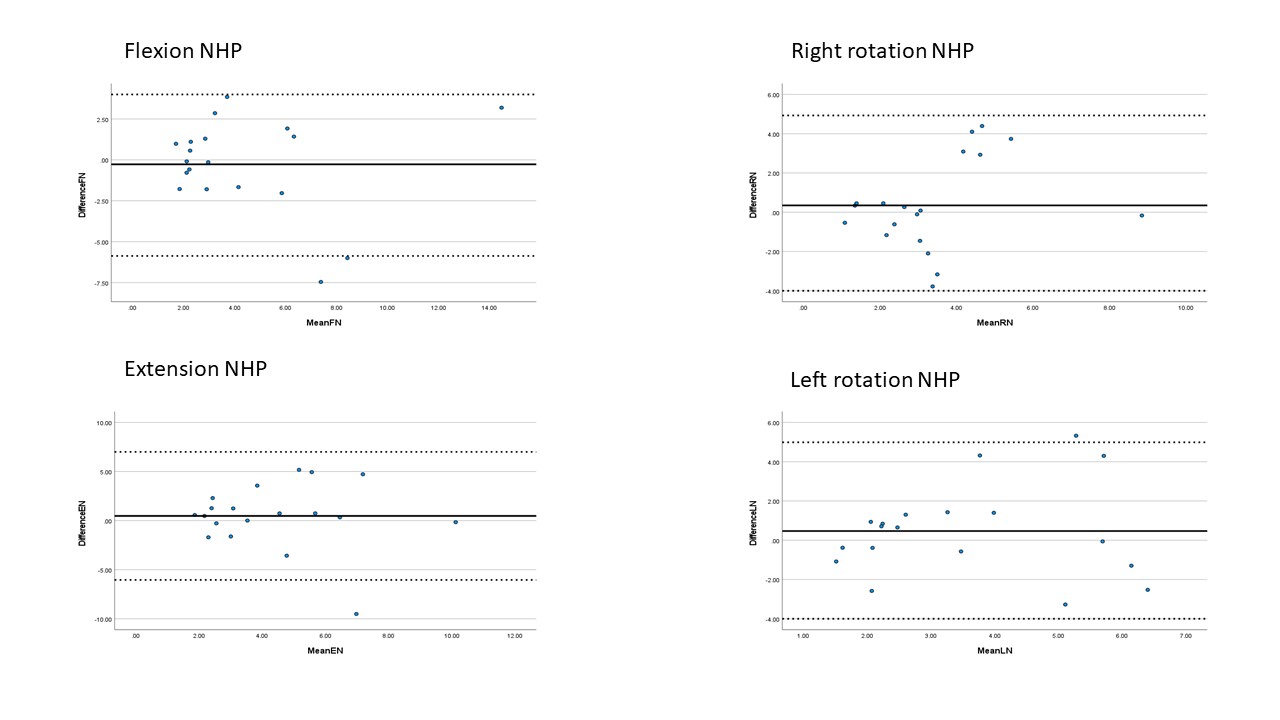


Figure 9, Bland Altman plots for intra-rater reliability of absolute error for flexion direction, extension, and right and left rotation for neutral head position (NHP) task in sitting. Limits of agreement are presented as the dotted lines with the mean difference illustrated by the black line.

Bland Altman plots for the limits of agreement for constant JPE measured in sitting show that most of the scores lie between the 95% confidence interval with mean differences -2.79-1.11 for NHP (Figure 10).


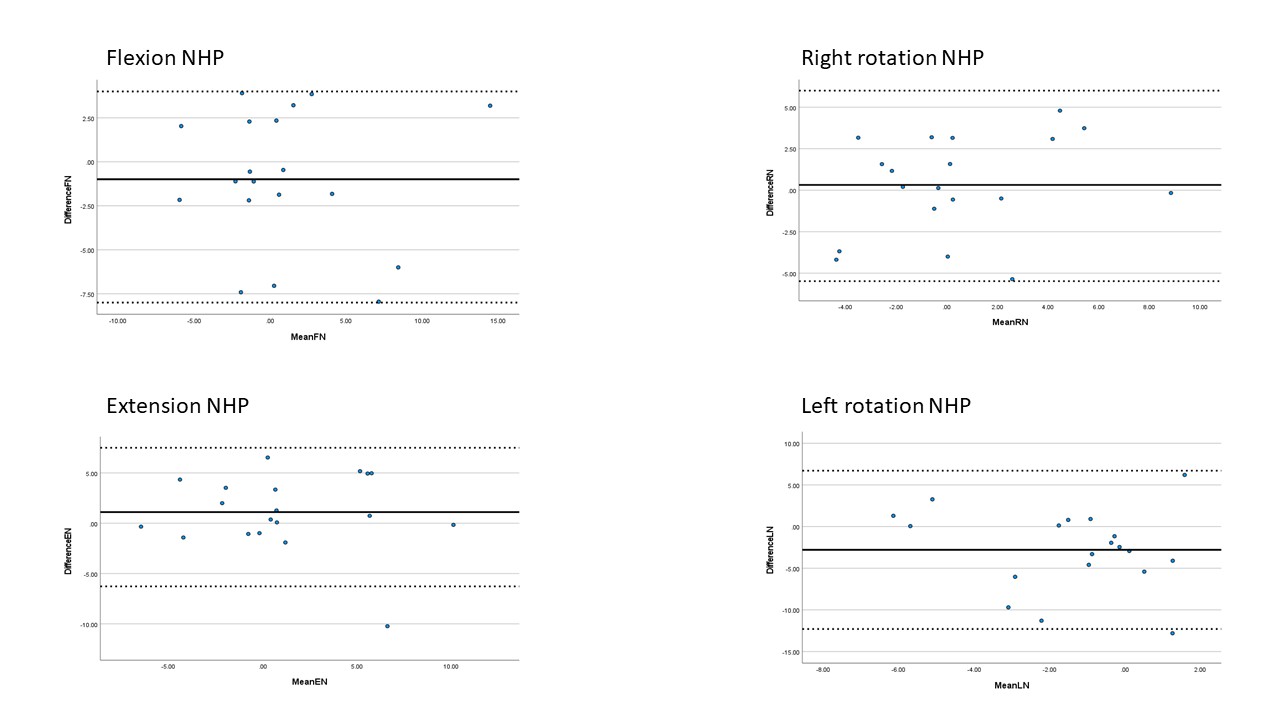


Figure 10, Bland Altman plots for intra-rater reliability of constant error for flexion direction, extension, and right and left rotation for neutral head position (NHP) task in sitting. Limits of agreement are presented as the dotted lines with the mean difference illustrated by the black line.

**Bland Altman plots for JPE in standing (CNP participants)**

Bland Altman plots for the limits of agreement for absolute JPE measured in standing show that most of the scores lie between the 95% confidence interval with mean differences -0.81-0.75 for NHP (Figure 11).


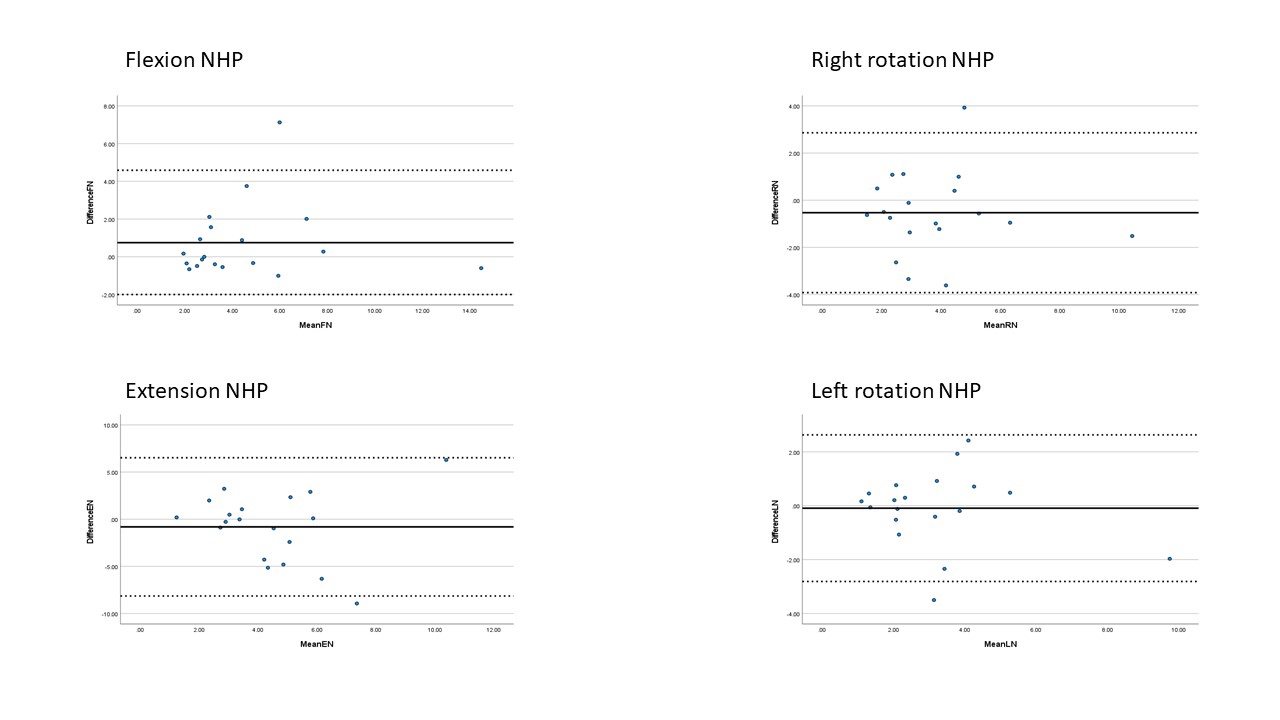


Figure 11, Bland Altman plots for intra-rater reliability of absolute error for flexion direction, extension, and right and left rotation for neutral head position (NHP) task in standing. Limits of agreement are presented as the dotted lines with the mean difference illustrated by the black line.

Bland Altman plots for the limits of agreement for constant JPE measured in standing show that most of the scores lie between the 95% confidence interval with mean differences -1.53-0.99 for NHP (Figure 12).


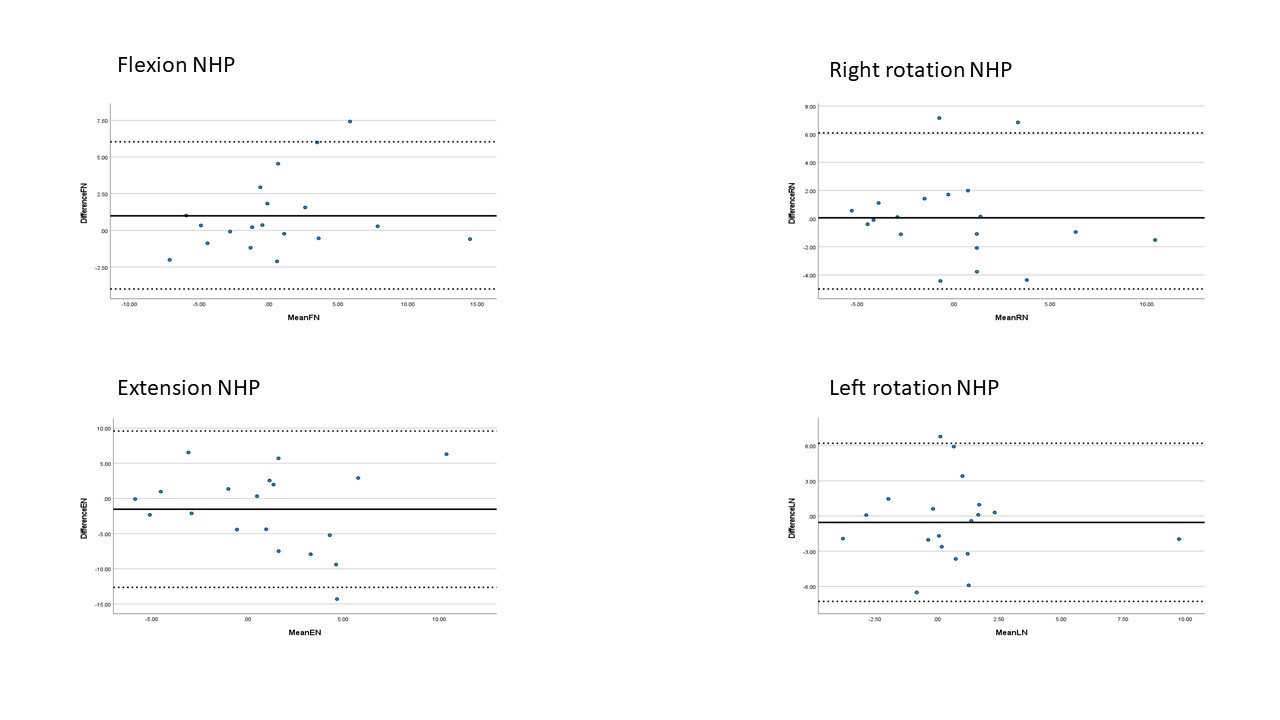


Figure 12, Bland Altman plots for intra-rater reliability of constant error for flexion direction, extension, and right and left rotation for neutral head position (NHP) task in standing. Limits of agreement are presented as the dotted lines with the mean difference illustrated by the black line.

**Validity**

**Bland Atman plots in sitting**

Bland Altman plots for the limits of agreement for absolute JPE measured in sitting show that most of the scores lie between the 95% confidence interval with mean differences -1.1 to 0.29 for the NHP (Figure 13) and 0.1-1.49 for the THP (Figure 14).


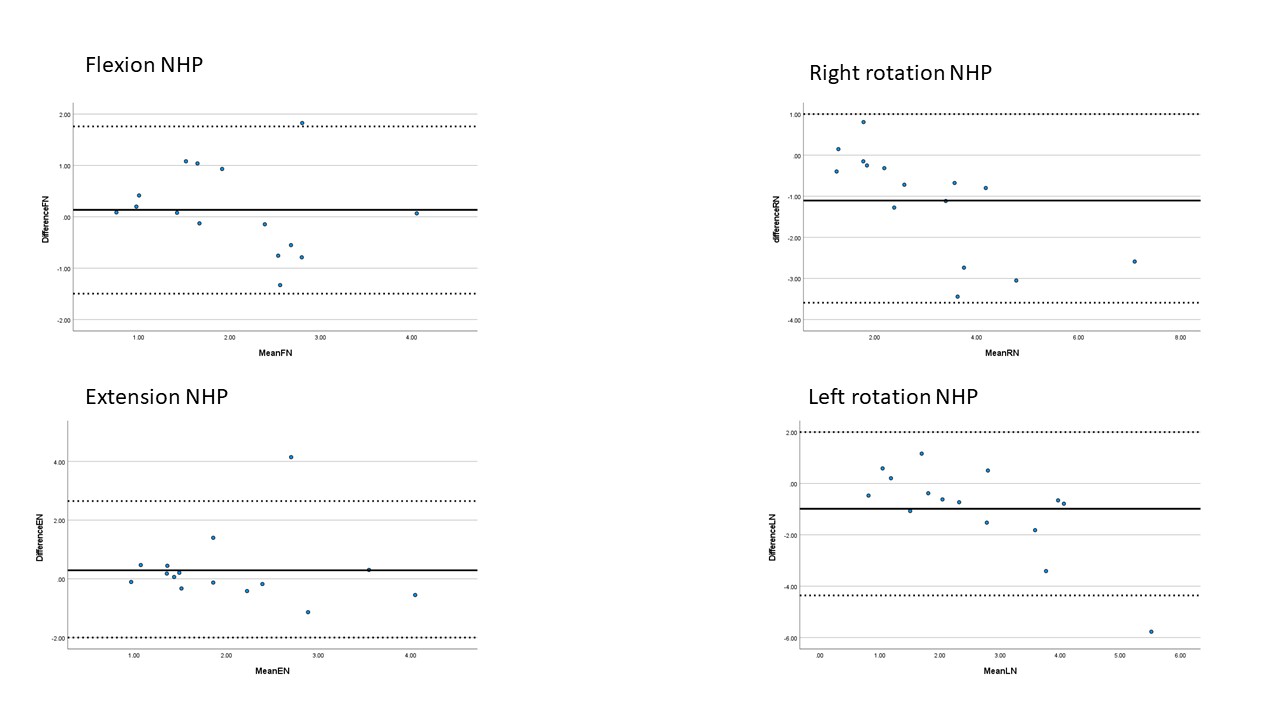


Figure 13, Bland Altman plots for criterion-related validity of absolute error for flexion direction, extension, and right and left rotation for neutral head position (NHP) task in sitting. Limits of agreement are presented as the dotted lines with the mean difference illustrated by the black line.


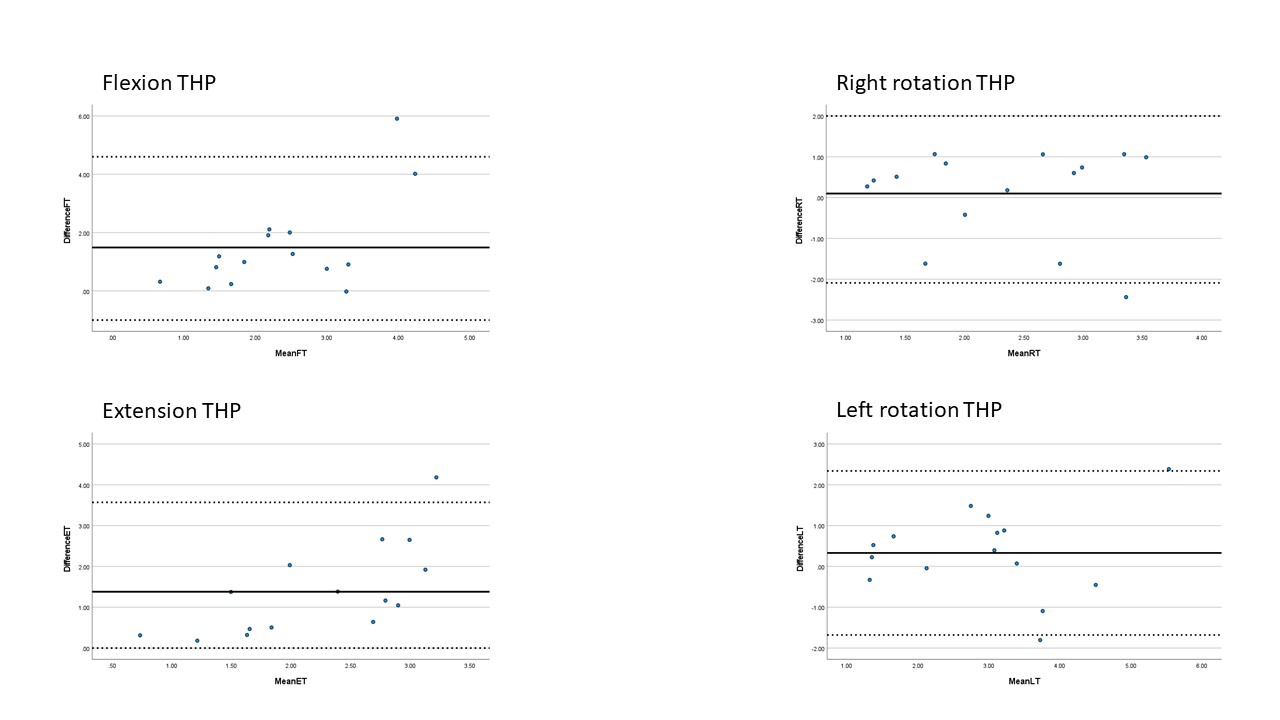


Figure 14, Bland Altman plots for criterion-related validity of absolute error for flexion direction, extension, and right and left rotation for target head position (THP) task in sitting. Limits of agreement are presented as the dotted lines with the mean difference illustrated by the black line.

Bland Altman plots for the limits of agreement for constant JPE measured in sitting show that most of the scores lie between the 95% confidence interval with mean differences -1.92-0.24 for the NHP (Figure 15) and -1.35-0.34 for the THP (Figure 16).


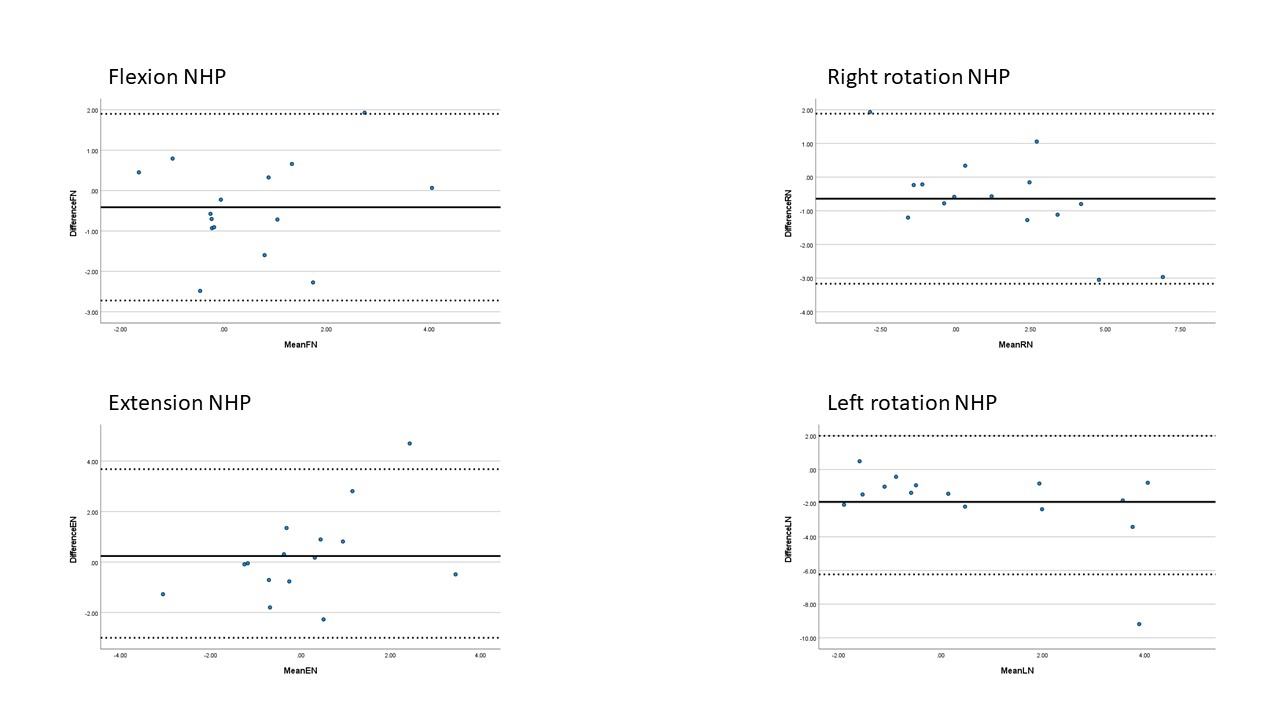


Figure 15, Bland Altman plots for criterion related validity of constant error for flexion direction, extension, and right and left rotation for neutral head position (NHP) task in sitting. Limits of agreement are presented as the dotted lines with the mean difference illustrated by the black line.


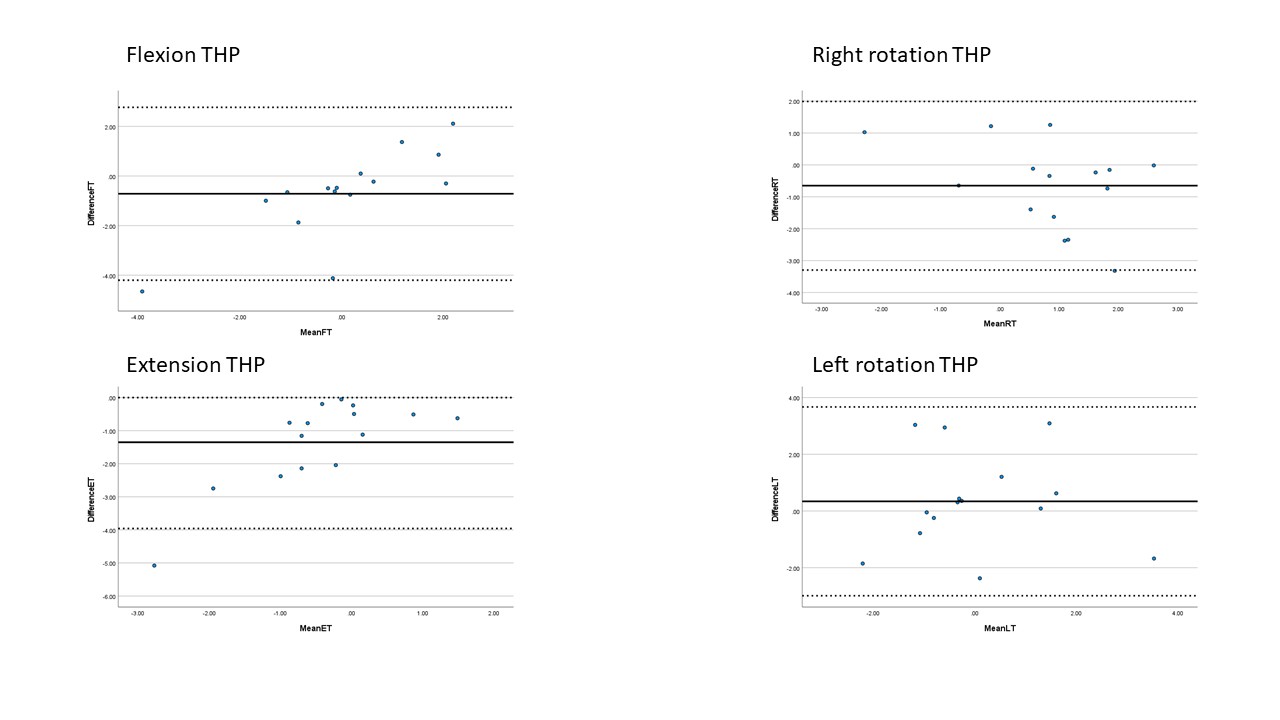


Figure 16, Bland Altman plots for criterion related validity of constant error for flexion direction, extension, and right and left rotation for target head position (THP) task in sitting. Limits of agreement are presented as the dotted lines with the mean difference illustrated by the black line.

**Bland Altman plots in standing**

Bland Altman plots for the limits of agreement for absolute JPE measured in standing show that most of the scores lie between the 95% confidence interval with mean differences -0.77 to 2.48 for the NHP (Figure 17) and -0.37 to 1.29 for the THP (Figure 18) apart from right rotation in NHP (Figure 17) where the scores are not evenly distributed above and below the mean difference indicating a bias.

**
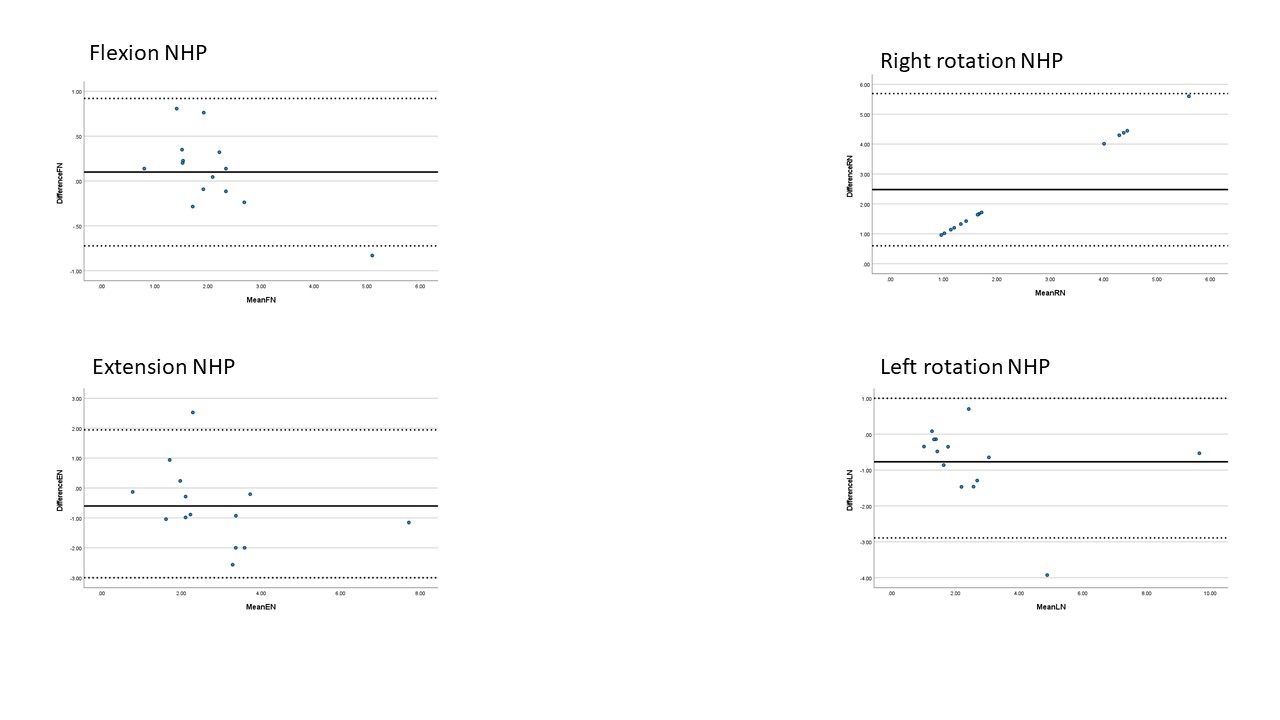
**

Figure 17, Bland Altman plots for criterion-related validity of absolute error for flexion direction, extension, and right and left rotation for neutral head position (NHP) task in standing. Limits of agreement are presented as the dotted lines with the mean difference illustrated by the black line.


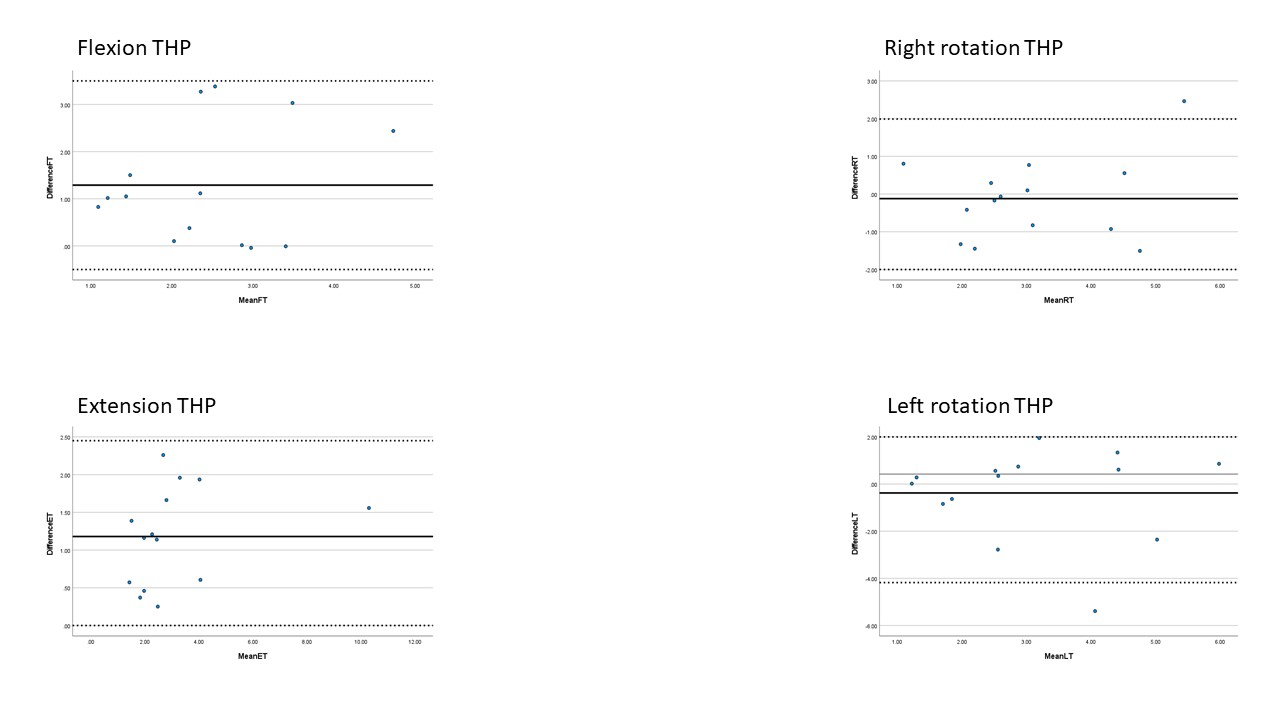


Figure 18, Bland Altman plots for criterion-related validity of absolute error for flexion direction, extension, and right and left rotation for target head position (THP) task in standing. Limits of agreement are presented as the dotted lines with the mean difference illustrated by the black line.

Bland Altman plots for the limits of agreement for constant JPE measured in standing show that most of the scores lie between the 95% confidence interval with mean differences -0.99 to 0.11 for the NHP (Figure 19) and -1.71 to -0.2 for the THP (Figure 20).


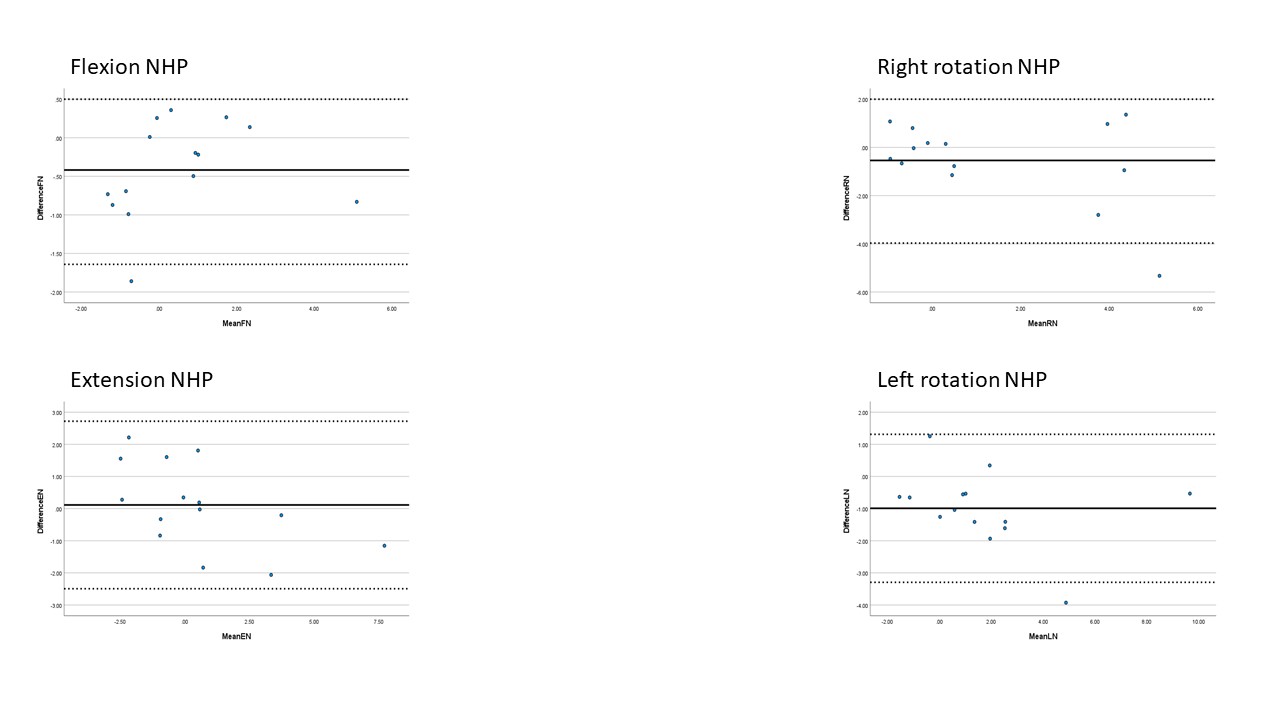


Figure 19, Bland Altman plots for criterion-related validity of constant error for flexion direction, extension, and right and left rotation for neutral head position (NHP) task in standing. Limits of agreement are presented as the dotted lines with the mean difference illustrated by the black line.


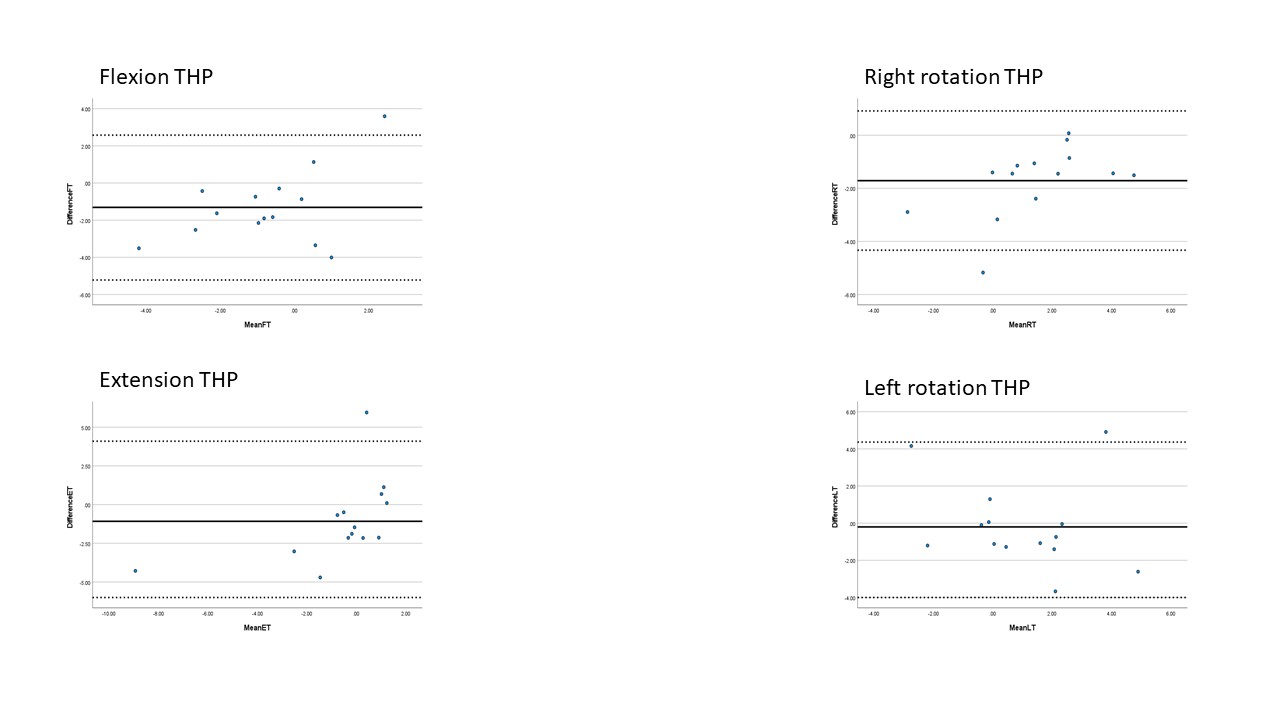


Figure 20, Bland Altman plots for criterion-related validity of constant error for flexion direction, extension, and right and left rotation for target head position (THP) task in standing. Limits of agreement are presented as the dotted lines with the mean difference illustrated by the black line.
